# Model-Based Quantification of the Systemic Interplay between Glucose and Fatty Acids in the Postprandial State

**DOI:** 10.1371/journal.pone.0135665

**Published:** 2015-09-10

**Authors:** Fianne L. P. Sips, Elin Nyman, Martin Adiels, Peter A. J. Hilbers, Peter Strålfors, Natal A. W. van Riel, Gunnar Cedersund

**Affiliations:** 1 Department of Biomedical Engineering, Eindhoven University of Technology, Postbus 513, 5600 MB, Eindhoven, The Netherlands; 2 Department of Biomedical Engineering, Linköping University, SE-58185, Linköping, Sweden; 3 CVMD iMED DMPK AstraZeneca R&D, 431 83, Mölndal, Sweden; 4 Health Metrics at Sahlgrenska Academy, University of Gothenburg, Göteborg, Sweden; 5 Department of Clinical and Experimental Medicine, Linköping University, SE-58185, Linköping, Sweden; Monash University, AUSTRALIA

## Abstract

In metabolic diseases such as Type 2 Diabetes and Non-Alcoholic Fatty Liver Disease, the systemic regulation of postprandial metabolite concentrations is disturbed. To understand this dysregulation, a quantitative and temporal understanding of systemic postprandial metabolite handling is needed. Of particular interest is the intertwined regulation of glucose and non-esterified fatty acids (NEFA), due to the association between disturbed NEFA metabolism and insulin resistance. However, postprandial glucose metabolism is characterized by a dynamic interplay of simultaneously responding regulatory mechanisms, which have proven difficult to measure directly. Therefore, we propose a mathematical modelling approach to untangle the systemic interplay between glucose and NEFA in the postprandial period. The developed model integrates data of both the perturbation of glucose metabolism by NEFA as measured under clamp conditions, and postprandial time-series of glucose, insulin, and NEFA. The model can describe independent data not used for fitting, and perturbations of NEFA metabolism result in an increased insulin, but not glucose, response, demonstrating that glucose homeostasis is maintained. Finally, the model is used to show that NEFA may mediate up to 30–45% of the postprandial increase in insulin-dependent glucose uptake at two hours after a glucose meal. In conclusion, the presented model can quantify the systemic interactions of glucose and NEFA in the postprandial state, and may therefore provide a new method to evaluate the disturbance of this interplay in metabolic disease.

## Introduction

Dysregulation in the postprandial handling of metabolites plays a central role in the development of metabolic diseases such as Type 2 Diabetes (T2D) and Non-alcoholic Fatty Liver Disease. This role can be recognized at a systemic level from the clear correlation between obesity and these metabolic diseases, as well as from the evidence that postprandial dysregulation of glucose or lipid metabolism (independently) predicts the risk of development of metabolic disease and its complications [1–8]. A good understanding of postprandial handling of metabolites at the systemic level is thus a prerequisite to understanding the development of metabolic diseases.

Knowledge on postprandial metabolite handling has been greatly expanded over the past few decades, due to application of stable isotope tracer and NMR spectroscopy techniques to study systemic and tissue-specific fluxes in glucose and lipid metabolism. In glucose metabolism, a gold standard of postprandial glucose flux measurement was established with the use of triple tracers [9], and quantification of the increase of postprandial hepatic glycogen stores have allowed quantification of hepatic glucose fluxes [10–13]. Untangling of postprandial lipid metabolism has benefited from stable isotope techniques that have revealed the origin of lipids in lipoprotein or non-esterified fatty acids (NEFA) pools [5,6,14–19]. However, because the response to a meal is dynamic, fast, and regulated by a wealth of hormonal and neurological mechanisms [4], the systemic interplay of mechanisms governing postprandial metabolite handling remains incompletely understood.

Plasma NEFA and glucose levels are tightly intertwined, and simultaneous dysregulation of both NEFA and glucose metabolism is associated with development of insulin resistance and hepatic lipid accumulation [20–24]. Since the initial proposition of direct interactions between glucose and NEFA metabolism in 1963 [24], a complex network of lipid–carbohydrate interactions in liver, adipose tissue, and skeletal muscle has been revealed [22,25], wherein NEFA acutely decreases hepatic insulin sensitivity [26–28] as well as peripheral insulin sensitivity [22,29,30]. The temporal and quantitative characteristics of these acute systemic NEFA-glucose interactions have been repeatedly investigated under clamp conditions where glucose and insulin levels are kept constant, and a triglyceride (TG) emulsion is infused [31–37]. However, the translation of such clamp measurements to a dynamic postprandial situation is difficult, since the concentrations of metabolites and hormones are both rapidly and simultaneously changing in the postprandial state. Therefore, we propose a mathematical modelling approach to handle such translational difficulties, and to gain insight into the systemic, postprandial interplay between glucose and NEFA.

Ordinary differential equation (ODE) models of the systemic, postprandial glucose-insulin interplay have been developed for several decades [38–42]. A hallmark model is the meal simulation model developed by Dalla Man *et al*. [41], which focusses on the postprandial state and incorporates gold standard systemic glucose flux data. This model has also proven to be of value as a simulation tool–e.g. in a Type 1 Diabetes simulator [39,43], and as the whole body component of a hierarchical modelling approach [44]. This meal simulation model does not, however, incorporate the kinetics of or interplay with NEFA.

For postprandial NEFA kinetics, several models have been introduced [42,45–47]. The interaction between NEFA and glucose metabolism, however, has not been included in these approaches, and therefore the effect of NEFA on glucose fluxes cannot be derived from these models. In summary, no existing model or experimental technique can currently elucidate the *in vivo* interplay of NEFA metabolism with the relevant glucose fluxes under postprandial conditions.

**Fig 1:**
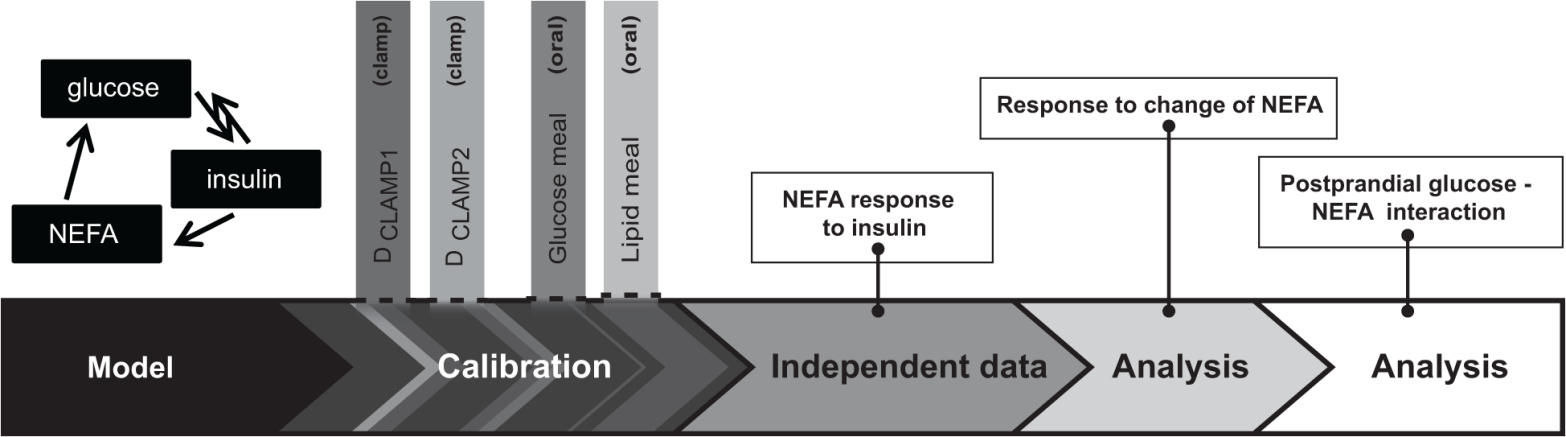
Overview. Overview of the workflow: model (Fig 2), model calibration with clamp simulations (Figs 3 and 4) and oral challenge simulations (Fig 5), comparison of the calibrated model with independent data (Fig 6), analysis of model response to NEFA perturbations (Fig 7), and quantification of NEFA regulation in the meal response (Fig 8).

Here, we thus develop a new model for the interplay of glucose, insulin, and NEFA at the systemic level. The model is based on the model of postprandial glucose-insulin interplay by Dalla Man *et al*. [41], and additionally includes NEFA kinetics, postprandial NEFA influx, and NEFA effects on glucose metabolism. The model is calibrated with data from literature: clamp data from two sources [32,33], and dynamic data describing the postprandial response to an intake of either lipid or glucose [48] (Fig 1). The quality of the developed model is tested by its ability to describe independent data, i.e. data not used for the calibration. Finally, the influence of NEFA on systemic glucose metabolism under postprandial conditions is quantitatively analyzed, thereby integrating the information available from the clamp experiments with a dynamic physiological situation.

## Materials and Methods

### Ordinary differential equation model

The mathematical model consists of a system of non-linear ordinary differential equations (ODEs) and can be represented as follows:

$$\begin{array}{l} \frac{\text{d}x}{\text{d}t}\;=\;f\left(x,p,u\right),\;x\left(0\right)=\;x_{0}\\ y\;=\;g\left(x,p\right) \end{array}$$ (1)

Where *t* is time (in minutes) and all other symbols represent vectors. The symbol *x* represents the states of the model, x_0_ denotes the initial values of these states, *p* are model parameters, *f* and *g* are non-linear functions, *u* represents the model inputs, and finally *y* are the model outputs which correspond to measurements.

### Model calibration

To calibrate an ordinary differential equation model, model simulations for values of the parameters *p* are compared to experimental data. More specifically, model calibration is performed by choosing the parameter values such that they best describe the experimental (calibration) data, i.e. by minimizing a cost function *V* (Eq 2). *V* is defined as the squared difference between model outputs (*y*_*i*_) and measured outputs (*y* ^*obs*^_*i*_), divided by the experimental variance (the square of the standard error *σ*_*i*_) to account for measurement uncertainty, and therefore represents the weighed sum of squared residuals.

$$V\left(p\right)\;=\;\sum \frac{{\left(y_{i}\left(p,t\right)-y^{obs}{}_{i}\left(t\right)\right)}^{2}}{\sigma_{i}{\left(t\right)}^{2}}$$ (2)

In this equation, *i* is the measurement index. During model calibration, an optimization procedure is employed to determine the optimal values of *p* (see below).

### Computer software

Data that was only available in a graphical format was digitized with the aid of PlotDigitizer 2.5.0 for Windows. The model was implemented and analyzed in MATLAB (MATLAB, Version R2012a, The MathWorks, Inc., Natick, Massachusetts, United States). The ordinary differential equation model was simulated using the variable step solver *ode15s*. Optimization and parameter sampling were performed from multiple starting points with a local, gradient-based least squares solver and a constrained scalar function solver (the built-in Matlab functions *lsqnonlin* and *fmincon*, respectively). Further information on settings and parameter boundaries can be found in S1 File.

### Mathematical model

**Fig 2:**
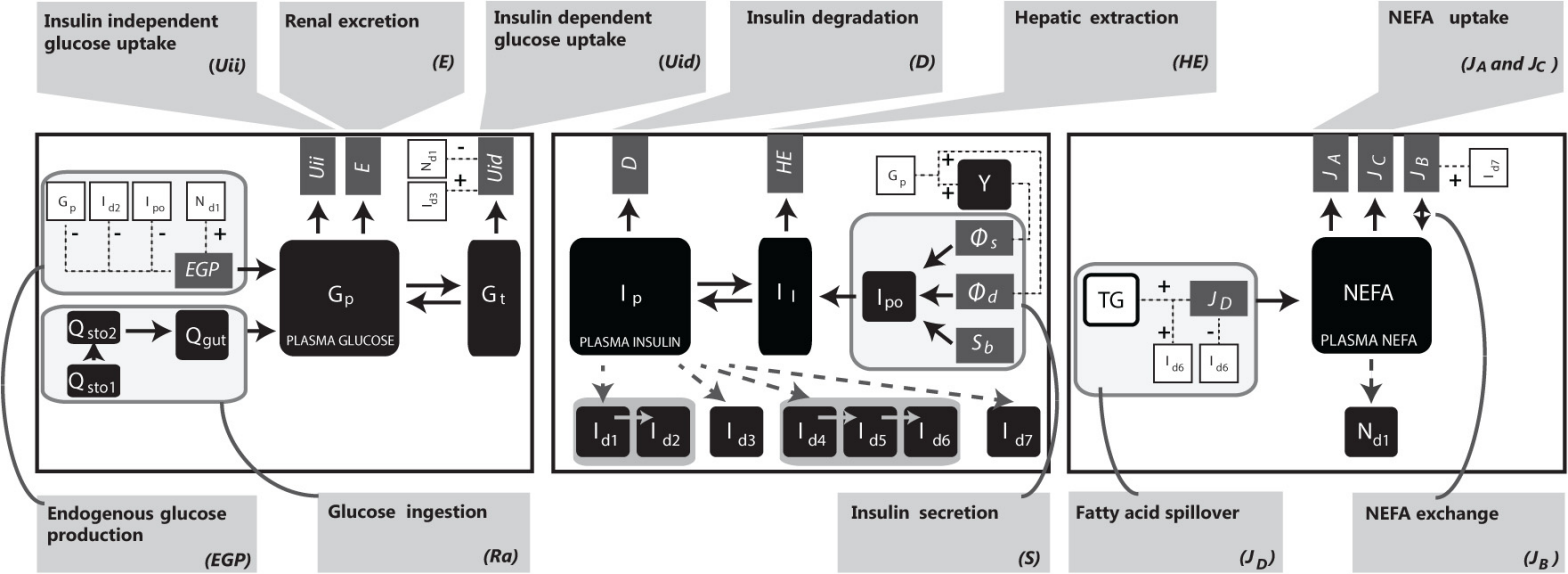
Mathematical model. The mathematical model of systemic glucose (left), insulin (middle) and NEFA (right) metabolism consists of a total of 18 differential equations. Glucose concentrations are determined by glucose rate of appearance (*Ra*), endogenous glucose production (*EGP*), insulin dependent (*U*_*id*_) and independent (*U*_*ii*_) glucose uptake and–if applicable–renal excretion (E). Plasma NEFA dynamics are described in Eqs 3–7. In the model, glucose enters the system via simulated ingestion in *Q*_*sto1*_, and lipid appearance is simulated by using the measured plasma TG concentration to calculate fatty acid spillover. For full model equations, we refer to S2 File. Matlab implementation and simulation files are provided as S3 File.

The detailed postprandial glucose-insulin model as published by Dalla Man *et al*. [41] served as a basis for the glucose and insulin equations. Similarly, the (fasting) NEFA kinetics are described by kinetic equations based on those published by Roy and Parker [49]. The NEFA kinetic equations were modified from [49] to (i) accommodate a non-steady state initial condition of NEFA [50], to (**ii**) remove the direct glucose regulation on NEFA dynamics which was based on *ex vivo* measurements [49], and (iii) to include adipose tissue spillover of NEFA (based on [42]). Herein, spillover represents the variable fraction of fatty acids released from lipoproteins via lipolysis that are released directly into circulation, instead of being taken up by the tissue. The full model is visualized in Fig 2.

Plasma NEFA is finally described by Eq 3.

$$\frac{\text{d}\;NEFA}{dt}=-J_{A}-J_{B}+J_{C}+J_{D}$$ (3)

Where *J*_*A*_, *J*_*B*_, *J*_*C*_ and *J*_*D*_ are given by Eqs 4–8. The term *J*_*A*_ represents the concentration dependent uptake of NEFA by the body, governed by parameter *p*_*A*_.

$$J_{A}=p_{A}\;NEFA$$ (4)

*J*_*B*_ represents insulin dependent inhibition of adipose tissue lipolysis, governed by parameter *p*_*B*_ and the difference between the insulin concentration in the remote compartment *I*_*d7*_ [49] and the basal insulin concentration *I*_*b*_.

$$J_{B}=p_{B}\;\left(I_{d7}-I_{b}\right)\;NEFA$$ (5)

*J*_*C*_ is a net flux that is assumed constant and smaller than zero in the parameterized model; thus, it represents a basal body fatty acid uptake;

$$J_{C}=p_{C}$$ (6)

Finally, *J*_*D*_ is the influx from the spillover of fatty acids released from plasma lipoproteins in the adipose tissue. The equation for *J*_*D*_ is composed of the absolute flux of fatty acids released from triglycerides in adipose tissue by insulin-stimulated LPL activity (*J*_*lpl*_), the insulin-inhibited ratio of fatty acids that are released via spillover (*spill*), and the distribution volume of plasma NEFA (*V*_*NEFA*_). Here, *spill* is the fraction of NEFA released from TG that spills into plasma, defined as an insulin-dependent fraction between zero and one.

$$J_{D}=\frac{spill\cdot J_{lpl}\left(TG\right)}{\begin{array}{c} V_{NEFA} \end{array}}$$ (7)

Postprandial TG extraction *J*_*lpl*_ *(TG)* was modelled using the insulin-regulated equation for lipoprotein lipase lipolysis of TG proposed by Jelic *et al*. [42], which requires measured systemic TG concentrations as a model input. The TG input is interpolated via a fitted polynomial function.

The hereby obtained modular model for glucose, insulin and NEFA was further expanded to account for the NEFA regulation of the glucose fluxes. More specifically, the endogenous glucose production (*EGP*) was changed to

$$EGP\left(t\right)=k_{egp1}-k_{egp2}\;G_{p}\left(t\right)-k_{egp3}I_{d2}\left(t\right)-k_{egp4}\;\left(I_{po}\left(t\right)+\frac{ins_{inf}\left(t\right)}{\gamma }\right)+k_{egp5}\;N_{d1}\left(t\right)$$ (8)

Where, as in Dalla Man *et al*. [41], *G*_*p*_ *(t)* is the plasma glucose content (mg/kg), *I*_*d2*_ *(t)* is a delayed insulin signal, and *I*_*po*_ *(t)* is the portal vein insulin concentration and represents insulin secretion. The modifications include the addition of *ins*_*inf*_ *(t)*, which represents the insulin infusion rate (pmol/kg/min) and is necessary to describe clamp experiments, and a delayed NEFA signal *N*_*d1*_ *(t)* [49]. The variable *k*_*egp1*_ is chosen such that *EGP*(0) = *EGP*_*b*_. Finally, *k*_*egp2*_, *k*_*egp3*_, *k*_*egp4*_, *γ* and *k*_*egp5*_ are model parameters.

Similarly, insulin-dependent glucose uptake (*U*_*id*_, from [41]) was re-formulated to include NEFA regulation as follows. *U*_*id*_ is described by

$$U_{id}\left(t\right)=\frac{V_{max,\;uid}\;\left(I_{d3}\left(t\right),\;N_{d1}\left(t\right)\right)\;G_{t}\left(t\right)}{K_{m,\;uid}\;+\;G_{t}\left(t\right)}$$ (9)

Where *G*_*t*_ is the tissue glucose concentration (*mg/dL*), *K*_*m*,*uid*_ is a model parameter and the *V*_*max*_ of the Michaelis-Menten expression is insulin- and NEFA dependent via Eq 10.

$$V_{max,\;uid}\;\left(I_{d3}\left(t\right),\;N_{d1}\left(t\right)\right)=k_{uid1}+k_{uid3}\frac{I_{d3}\left(t\right)}{N_{d1}\left(t\right)}$$ (10)

Here, *k*_*uid1*_ and *k*_*uid3*_ are again model parameters, *I*_*d3*_ is a delayed insulin signal and *N*_*d1*_ is a delayed NEFA signal; implemented to represent the observed reciprocal relationship between NEFA concentrations and insulin-dependent glucose uptake.

The complete model describes the systemic pre- and postprandial kinetics of glucose, insulin and NEFA and is outlined in Fig 2. Implementation details, full model equations, and software are provided as S1 File, S2 File, and S3 File respectively.

### Calibration data

To find values for the 21 free model parameters, a calibration dataset was composed by combining data from three sources [32,33,48]. All data were digitized from plots in the publications, and hence the model describes the population mean (as do the models in [41,42,49]).

The first two datasets, *D*_*CLAMP1*_ and *D*_*CLAMP2*_, describe the endogenous glucose production and insulin-dependent glucose uptake in healthy subjects in response to a variety of clamped conditions. These two datasets were chosen to obtain variability in both the time-scales of the experiments, as well as the fixed concentrations of glucose and insulin. The final dataset (*D*_*MEAL*_) describes the response of plasma glucose, insulin, NEFA and TG of a group of young, healthy men to both an oral glucose tolerance test (OGTT) and an oral lipid tolerance test (OFTT). This third dataset was chosen for its combination of isolated responses to glucose and lipids. Taken together, these data allow for estimation of the unknown parameters.

### Clamp-datasets

**Fig 3:**
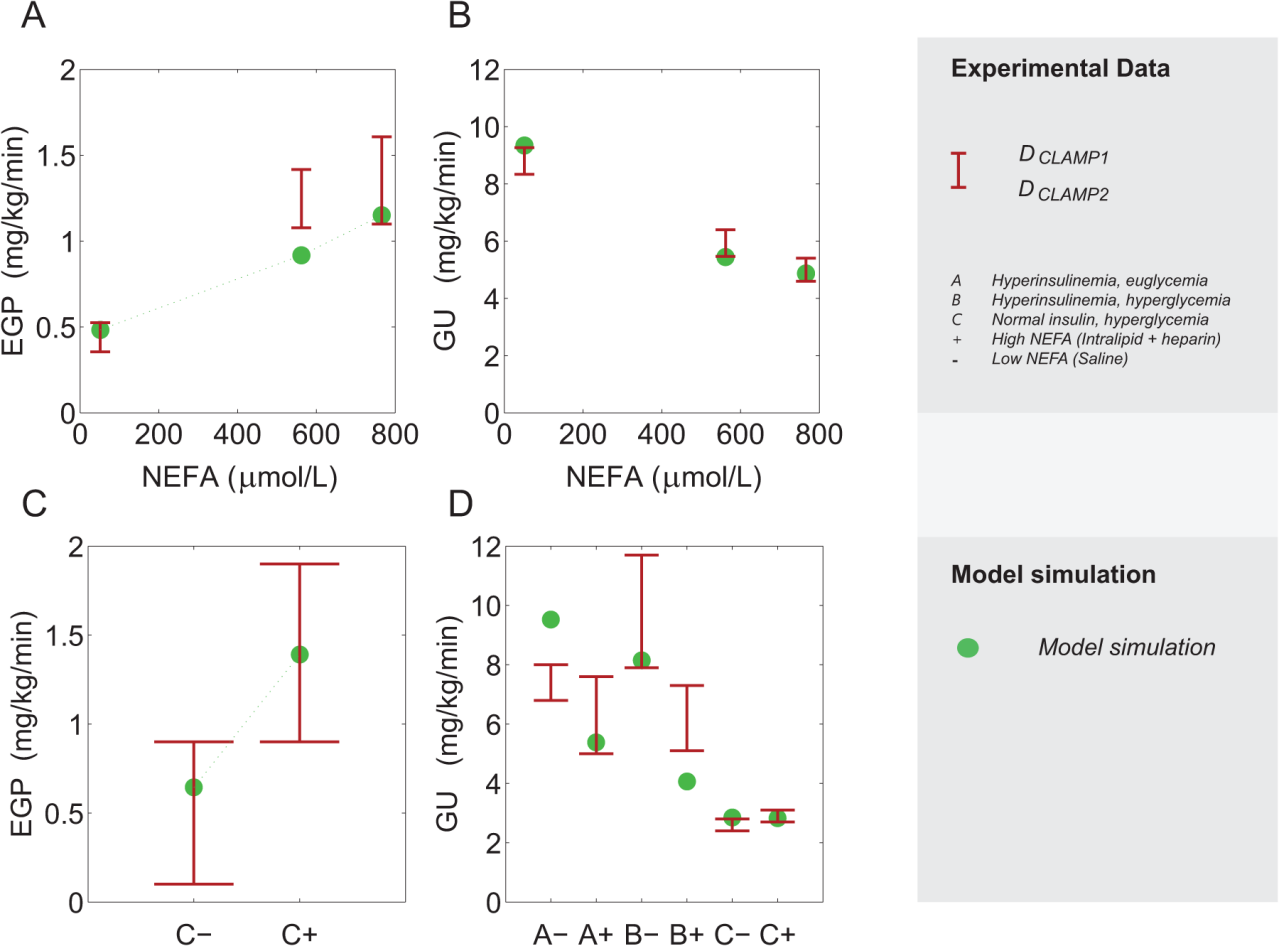
Clamp datasets: experimental data and simulation results. Measurements from D_CLAMP1_ (hyperinsulinemic, euglycemic clamp) **[32]** (A,B, red errorbars) and D_CLAMP2_ **[33]** (C,D, red errorbars) with superimposed model outputs. The simulation represents the parameter set in S_sel_ that corresponds to a minimal value for V_EGP_. A. Mean EGP as measured over the final half of a 360 minute clamp with low, medium and high NEFA concentration. B. Total glucose uptake (conditions and measurement time as in A). C. EGP measured during the final 60 minutes of the 120 minute clamp in experiments of group C that underwent an eu-insulinemic, hyperglycemic clamp with a saline infusion (C-) and with a combined intralipid and heparin infusion (C+). D. Total glucose uptake as in C, for experiments with a hyperinsulinemic euglycemic clamp (group A-, A+), hyperinsulinemic, hyperglycemic clamp (group B-, B+), and an eu-insulinemic, hyperglycemic clamp (group C- and C+). A short summary of the implementation in the model is provided in the Materials and Methods; full details of implementation can be found in S1 File.

**Fig 4:**
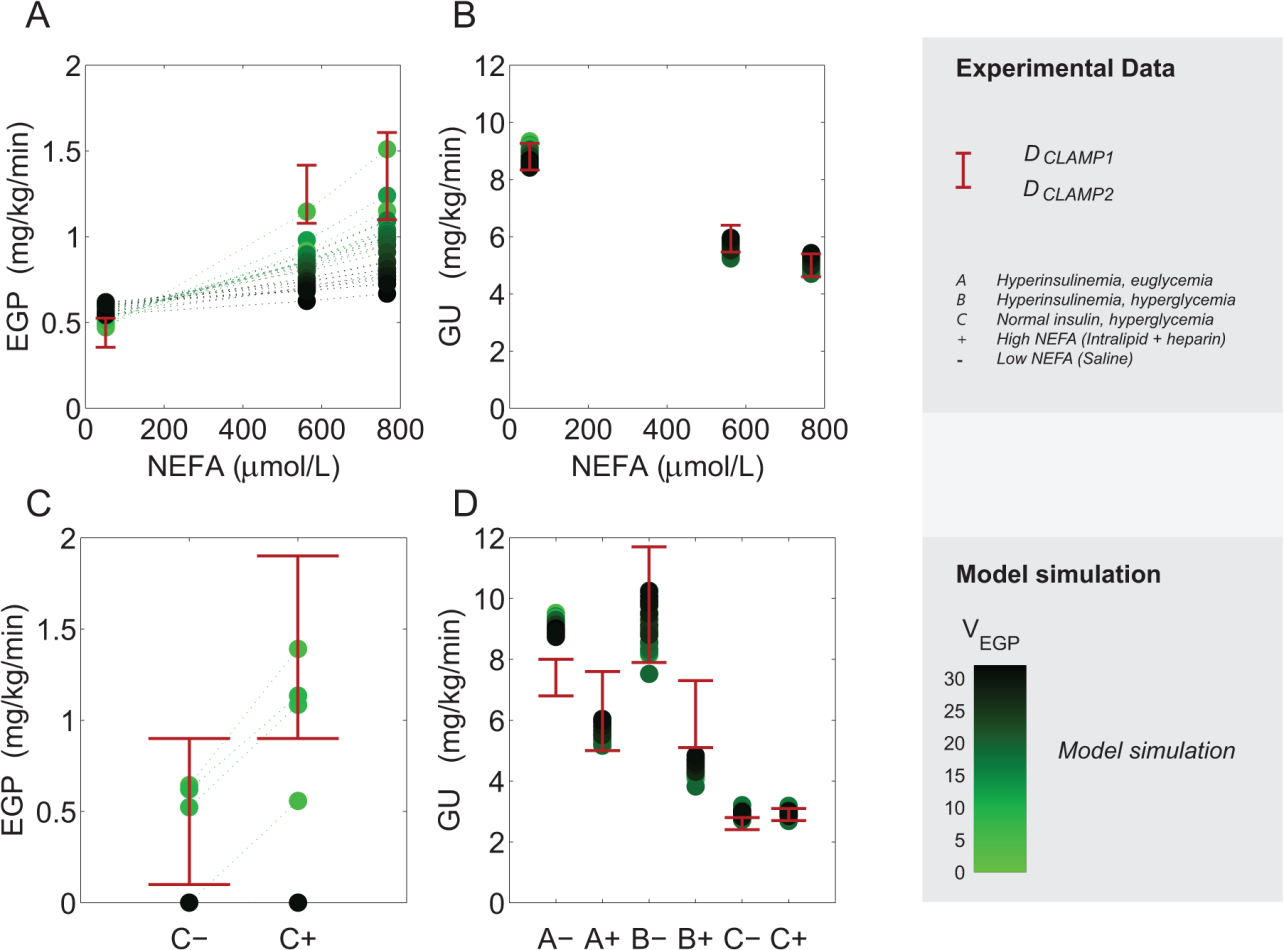
Model calibration on clamp data. To investigate propagation of parameter uncertainty in predictions and analyses a collection of parameter sets was selected. Measurements from D_CLAMP1_ (hyperinsulinemic, euglycemic clamp) **[32]** (A,B, red errorbars) and D_CLAMP2_ **[33]** (C,D, red errorbars) with superimposed model outputs as in Fig 3. Here, simulations representing the complete collection of selected parameter sets (S_sel_) are shown, depictured as dots shaded from dark green for poor fits of EGP (high values of V_EGP_) to light green for low V_EGP_. We note in C, that not all parameter sets from S_sel_ describe the data, and that a bad correspondence of the simulations in A and C is shown with dark green color.

For the first clamp data set (*D*_*CLAMP1*_, Figs 3, 4A and 4B), three groups of subjects (n = 4 for group A, n = 4 for group B and n = 6 for group C) underwent a hyperinsulinemic (70 μU/mL), euglycemic (85 mg/dL) clamp. During this clamp, subjects of group A received a continuous infusion of a TG emulsion and heparin, subjects of group B received only the TG emulsion and subjects of group C underwent a vehicle (saline) infusion. In group A, heparin is used to stimulate lipoprotein lipase activity, resulting in a larger increase of NEFA than in group B. The NEFA concentrations in the three groups were measured to reach three different plateaus of NEFA concentration–at means of 766, 562 and 51 μM respectively. The clamps were maintained for 6 h, and in the final 3 h of the study EGP and glucose uptake (GU) were calculated from stable isotope tracer kinetics. For full experimental methods and results we refer to the original publication [32].

For the second clamp data set (*D*_*CLAMP2*_, Figs 3, 4C and 4D), subjects were again divided into three groups (n = 6, 7 and 7 for A, B and C respectively); however, here the subjects in each group underwent a clamp on two different occasions. On one occasion, the subjects received a simultaneous infusion of a TG emulsion with heparin, while on the other occasion the infusion only contained vehicle. In each group, the settings of the clamp were designed to represent a different combination of hyper or eu-glycaemia and hyper- or eu-insulinemia. Group A underwent a hyperinsulinemic (raised by 100 μU/mL), euglycemic clamp; group B underwent a hyperglycemic (200 mg/dL), hyperinsulinemic clamp (50 μU/mL) and group C underwent a protocol in which glucose concentrations were raised (300 mg/dL) under relatively normal insulin concentrations. Each clamp was maintained for 2 hours, and EGP and GU were determined during the final hour. EGP was only determined in all subjects in group C, and for this reason, and because insulin infusion rates (necessary to simulate the clamp in the model) in group A were not reported, only EGP measurements from group C were available for inclusion in the dataset. For a full description of experimental methods and results we refer to the original publication [33].

Briefly, simulation of these clamp datasets is performed by fixing the main plasma states of the plasma glucose content *G*_p_ (mg/kg), the plasma insulin content *I*_*p*_ (pmol/kg) and the plasma NEFA concentration *NEFA* (μmol/L), as well as the exogenous insulin appearance and the time-derivative of glucose, to the values reported. In the first dataset (*D*_*CLAMP1*_), the plateau values of the clamp were used to define the NEFA concentration during the simulation. In the second clamp dataset (*D*_*CLAMP2*_), however, the concentration of NEFA did not reach a clear plateau and instead the concentration was described by the dynamic NEFA response, which was digitized and interpolated linearly. In each case, an average value for EGP and GU was calculated as in the experimental protocol, and the average value was compared to the measured value. Both clamp-datasets together contain five EGP measurements (three in *D*_*CLAMP1*_ and two in *D*_*CLAMP2*_ *)* and nine glucose uptake measurements (three from *D*_*CLAMP1*_ and six from *D*_*CLAMP2*_).

### Oral-challenge dataset

**Fig 5:**
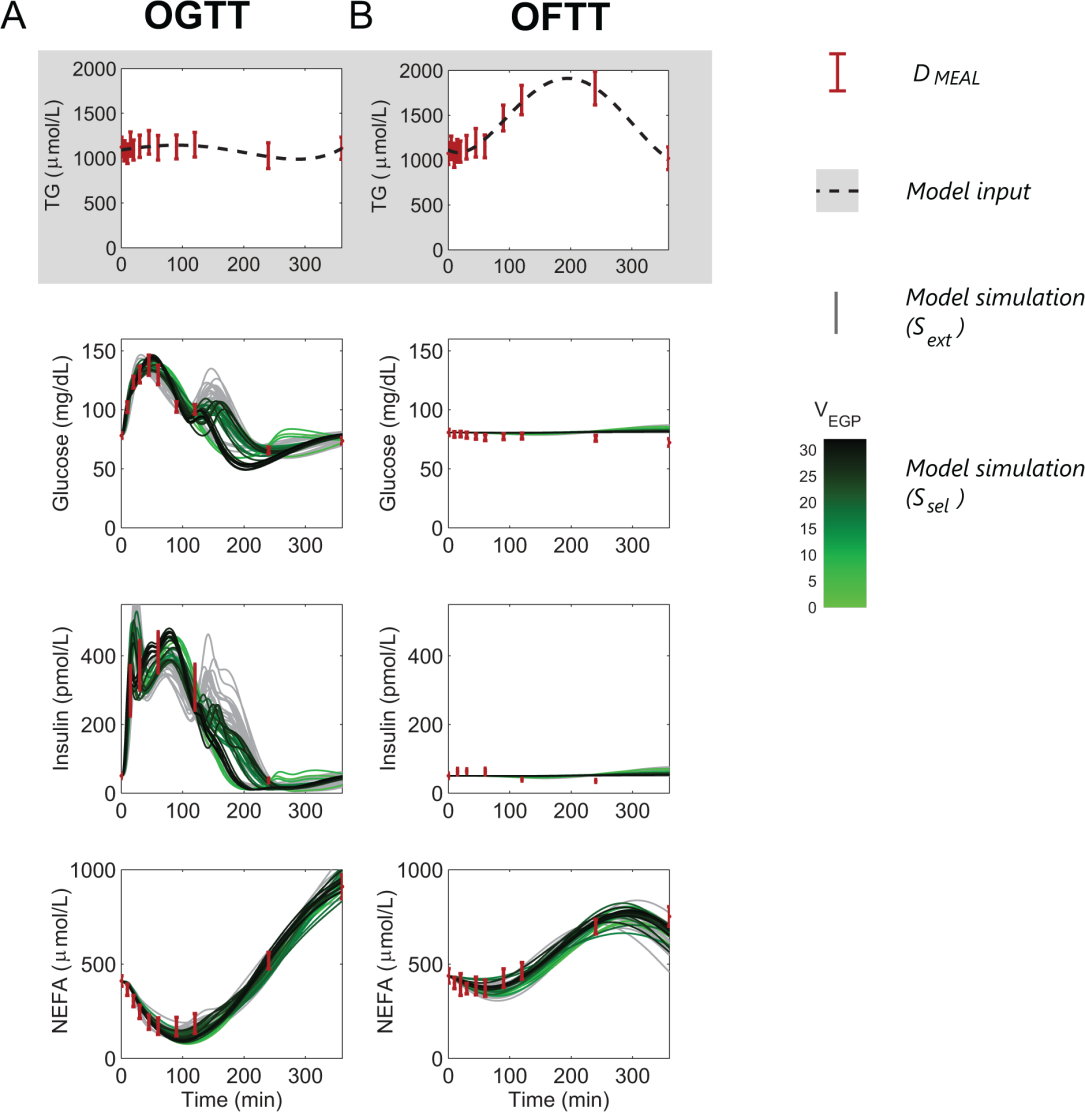
Model calibration on meal responses. Data from D_MEAL_ **[48]** (red errorbars) and model simulations (S_ext_, grey curves; and S_sel,_ green shading as in Fig 4) in response to an OGTT (A) and an OFTT (B).

The oral-challenge data D_MEAL_ was obtained from Robertson *et al*. [48]. It describes the response of 12 healthy male subjects to oral glucose (OGTT, 100 grams of glucose) and lipid (OFTT, cream containing 40 grams of fat), measured on two separate occasions. Plasma glucose, insulin, NEFA and TG were measured with high time resolution, especially in the beginning of the protocol (Fig 5). Measurements continue for 6 hours and include the plasma NEFA overshoot. The oral challenges were consumed in the morning, following an overnight fast preceded by a high-carbohydrate evening meal. For a full description of experimental methods and results we refer to [48].

Simulation was performed with inputs of glucose into the stomach compartment. Plasma TG concentrations were set to experimentally determined values, using a polynomial for interpolation.

### Optimization and parameter sampling

The model was calibrated to the entire calibration dataset (*D*_*MEAL*_, *D*_*CLAMP1*_ and *D*_*CLAMP2*_) as described in Eq (2). Most parameters were fixed to the values reported in [41,42,49,51], while remaining free parameters were estimated based on the calibration data. Free parameters are parameters with no previous known value or parameters presumed to be modified from the original value as a result of changes to the model or the conditions (See S1 File).

To investigate propagation of parameter uncertainty in predictions and analyses a collection of parameter sets was selected (using a method based on Step 1 in [52]). First, parameter sets yielding a cost function value within 120% of the lowest obtained cost function value were selected from a large parameter sampling obtained during optimization procedures. Parameter sets within this collection with extreme values of each parameter were chosen for visualization, this selection of parameter sets is referred to as *S*_*ext*_. For some parameter sets in *S*_*ext*_, however, the obtained value of the minimal stomach emptying parameter (*k*_*ra3*_) was equal to or lower than the value determined for a mixed meal [41,51]. As has been shown previously, this parameter should have a higher value for an OGTT than for a mixed meal [51]. Therefore, a second selection for the parameter sets was undertaken. The selection S_sel_ contains the extremes of the parameter sets that (1) have a cost function value within 120% of the lowest obtained cost function value and (2) have a value of at least 0.009 min^-1^ for *k*_*ra3*_ (further information can be found in S1 File).

**Fig 6:**
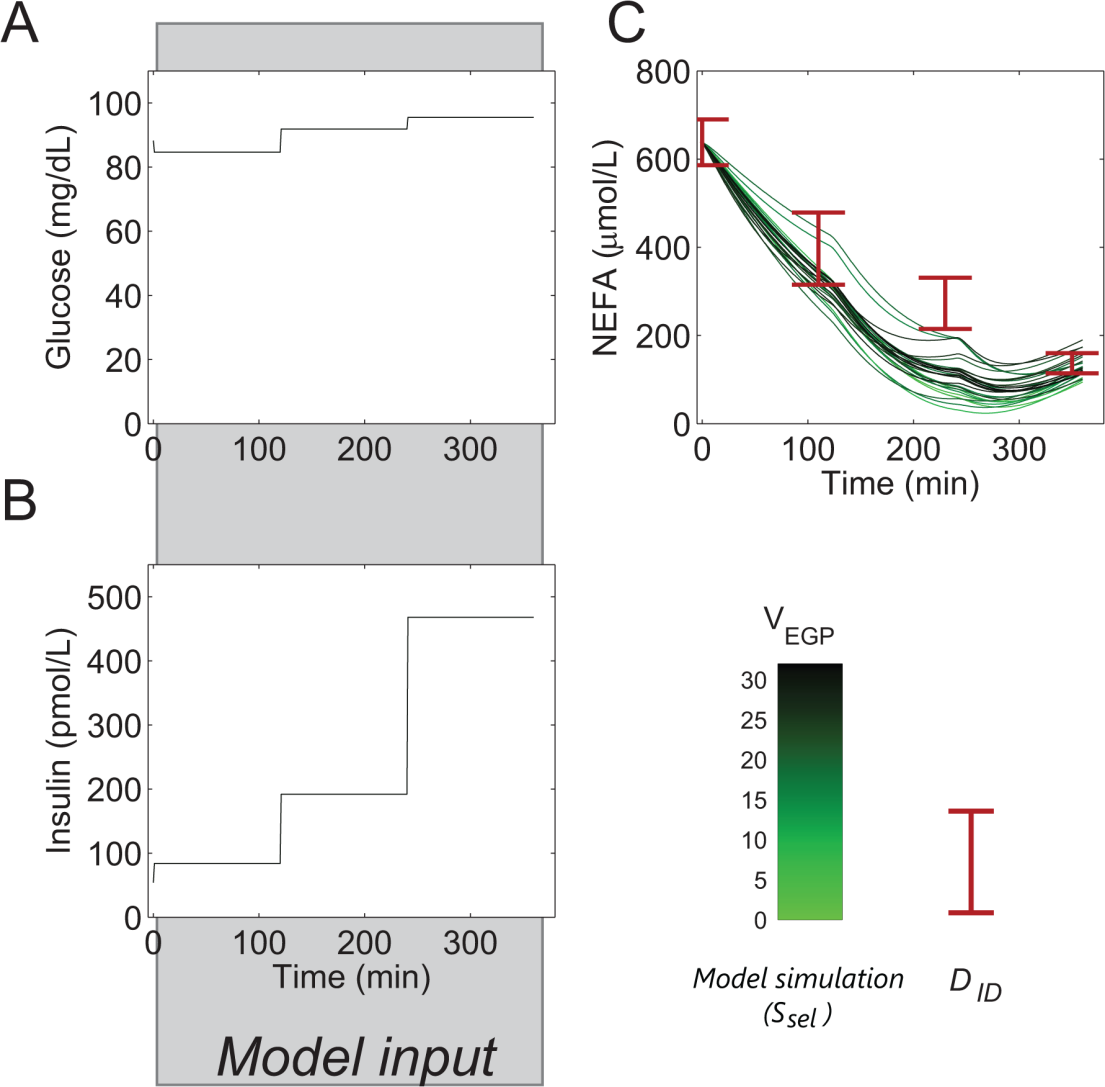
Model prediction–NEFA kinetics during a multi-step insulin infusion. To verify general NEFA kinetics, a multi-step insulin infusion (Campbell *et al*. **[53]**) was modelled by fixing glucose (A) and insulin (B) to clamped concentrations. As TG was not measured, it was fixed at 1000 μM. The initial NEFA concentration was fixed at the measured value. (C) Simulated NEFA concentration, S_sel_ (shades of green, as in Fig 4) and measurements of the independent clamp dataset **[53]** (red errorbars).

**Fig 7:**
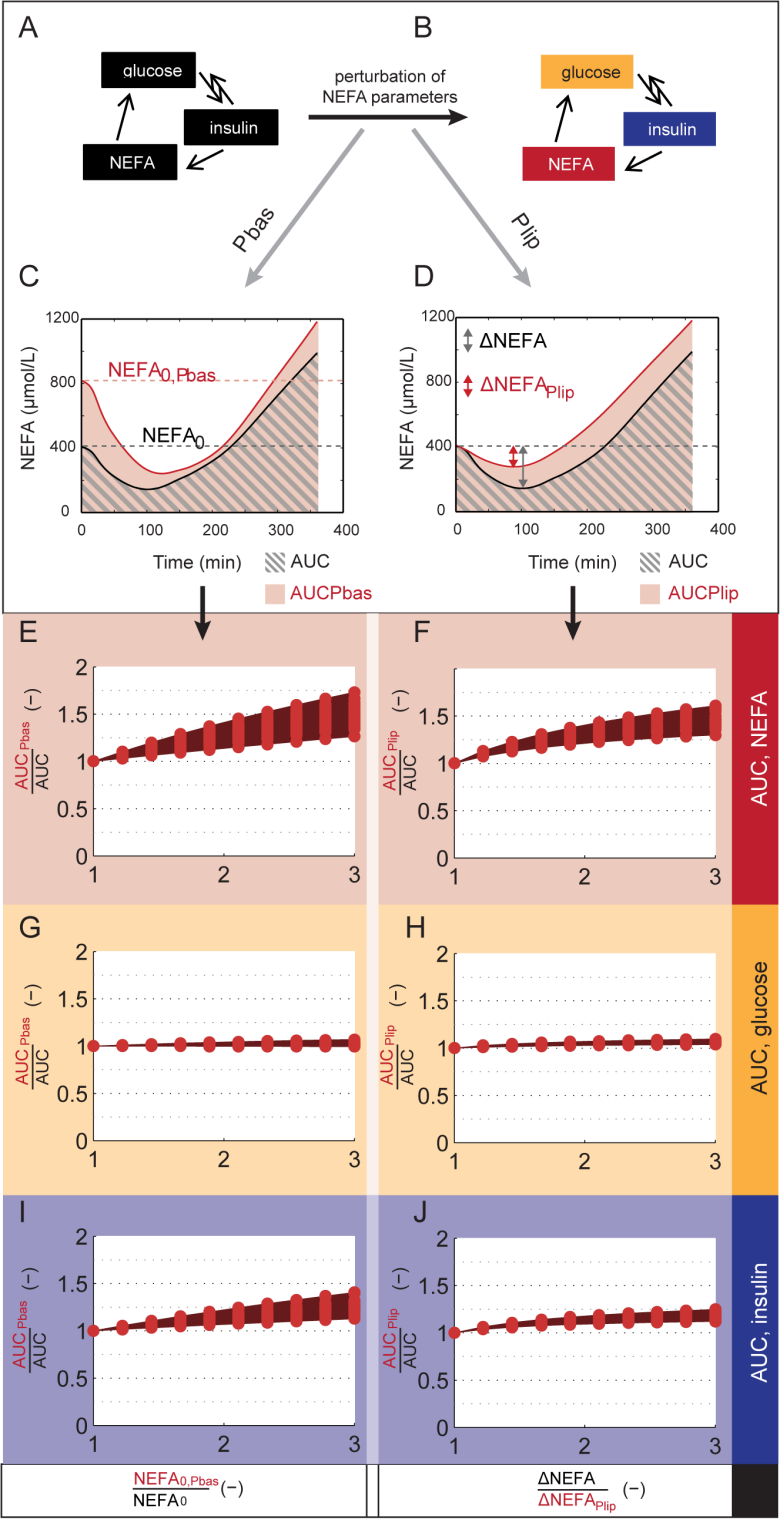
Model analysis–glucose homeostasis. Perturbations of NEFA metabolism affect the AUC of the glucose, insulin and NEFA responses to the OGTT. A-D. Overview of perturbation strategy. A. Original OGTT response model. B. Perturbed model. C. Perturbation by increasing the initial NEFA concentration (P_bas_ in the illustration). D. Perturbation by reducing the insulin dependent inhibition of lipolysis (P_lip_ in the illustration). E-G-I. Relative change in AUC of NEFA (E), glucose (G) and insulin (I) in response to an increase in the initial concentration of NEFA. F-H-J. Relative change in AUC of NEFA (F), glucose (H) and insulin (J) in response to reduced insulin-dependent inhibition of lipolysis. Red markers represent individual simulation results per parameter set; the shaded area (dark red) gives the full range.

**Fig 8:**
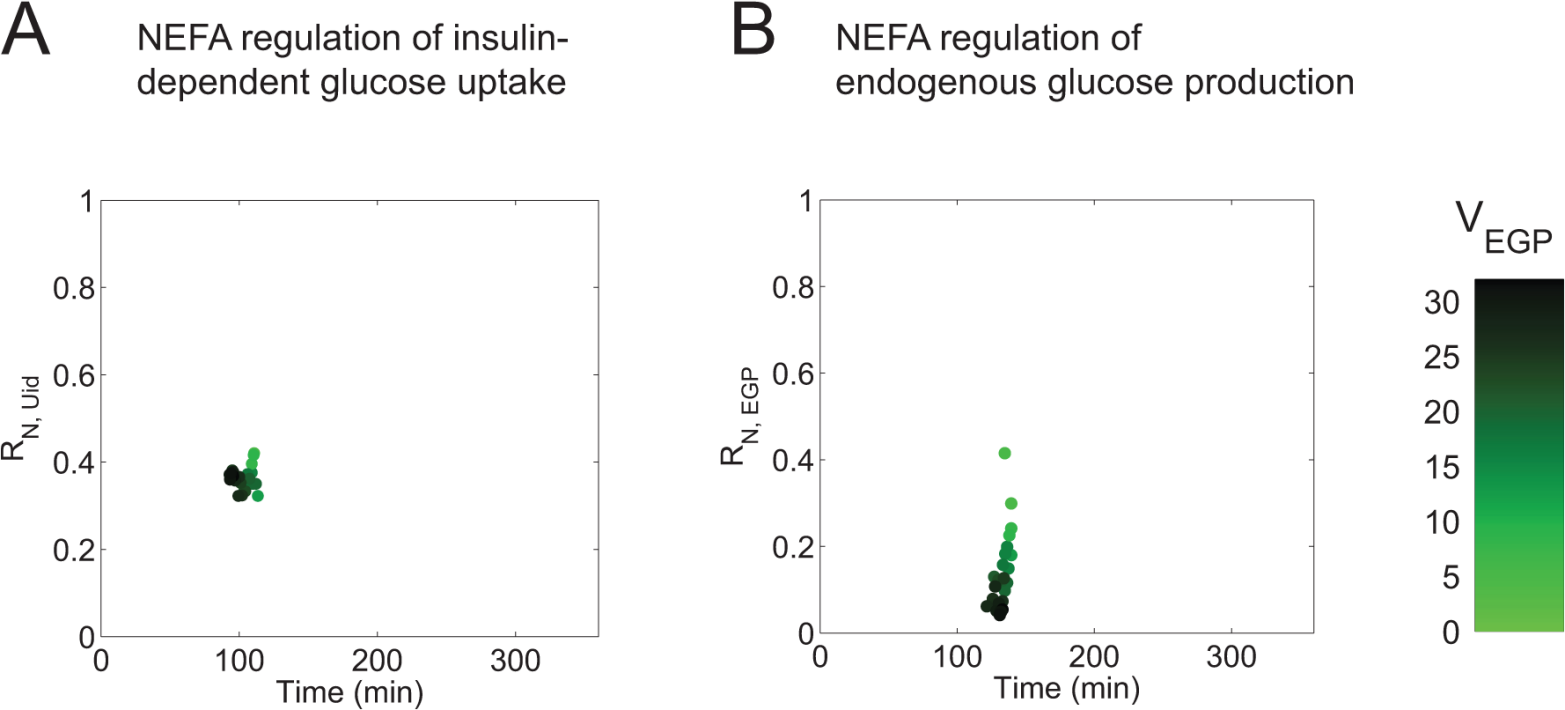
Model analysis—contribution of NEFA regulation in the postprandial glucose response. A. Relative contribution of regulation by the NEFA-regulation term N_d1_(t), on U_id_ during an OGTT, plotted at the time point where the absolute value of the NEFA regulation of U_id_ is maximal. B. Relative dependence of EGP suppression on the regulation by NEFA, plotted at the time point when the absolute NEFA-induced suppression is maximal. Colors indicate V_EGP_ (green shading as in Fig 4). The individual results represent the parameter sets in S_sel_.

To visualize the propagation of a good or bad fit to the EGP data in the remaining analyses, the cost function value was also calculated for (only) the five EGP data points in Fig 4A and 4C. This value is referred to as *V*_*EGP*_ and is used for visualization in Figs 4–8 (shades of green).

### Analysis of perturbations in NEFA metabolism

To examine the behavior of the model in response to acute changes of NEFA metabolism, we investigated how perturbations of postprandial NEFA dynamics affected the OGTT response simulation of *D*_*MEAL*_. Perturbation of NEFA metabolism was simulated in two ways: through elevation of fasting NEFA concentrations (*P*_*bas*_) and by reduction of the insulin sensitivity of lipolysis (*P*_*lip*_). In both cases, the perturbation of NEFA metabolism is performed by choosing a different value for a single parameter of NEFA metabolism. To analyze the effects of the perturbation on the system, the change of the area under the curve (AUC) of plasma glucose, insulin and NEFA concentrations during an OGTT was then determined. These perturbations yield a higher AUC for NEFA by design, but only affect glucose and insulin kinetics and AUC indirectly.

In the first perturbation *P*_*bas*_, the OGTT simulation was repeated after changing the fasting concentration of plasma NEFA (*NEFA*_*0*_). In the second perturbation *P*_*lip*_, the initial concentration of NEFA was not changed. Instead, simulations of the OGTT were performed in which the characteristic postprandial insulin-mediated fall of the concentration of NEFA was decreased. This was achieved by tuning the value of the insulin-dependent lipolysis parameter (*p*_*B*_) to the desired reduction of the postprandial fall of the concentration of NEFA. The model was then simulated with all parameters except for *p*_*B*_ at their original value.

### Analysis of the contribution of NEFA to glucose regulation

To evaluate the contribution of NEFA-mediated regulation of postprandial glucose metabolism, the following quantitative analysis was performed. First, the regulation of *U*_*id*_ and *EGP* during the OGTT (*D*_*MEAL*_) was divided into (1) the non-NEFA regulated contributions and (2) the NEFA regulation terms. Then the NEFA regulation term was divided by the sum of all regulatory terms to obtain the relative contribution of NEFA to the total regulation of insulin-dependent glucose uptake *R*_*N*,*Uid*_ *(t)* and the relative contribution of NEFA to the total regulation of endogenous glucose production *R*_*N*,*EGP*_ *(t)*. The calculation of these variables is difficult when the total regulation approaches zero, and therefore only the maximal values of these regulations are selected for plotting. Equations and details of the implementation can be found in the S4 File.

## Results

We developed a new mathematical model for the dynamic interplay of NEFA with insulin and glucose metabolism, as detailed in Materials and Methods. The new model is calibrated against published data from three sources: data of the NEFA-dependent hyperglycemic and/or hyperinsulinemic clamp response from [32,33], and data of the dynamic postprandial response from [48]. The calibration of the model is visualized in Figs 3–5.

### Simulation of glucose response to different clamp conditions

Fig 3 shows the model output for a parameter set that describes the calibration data adequately, including EGP measurements of the clamp datasets (Fig 3A and 3C). Hereto the parameter set in S_sel_ (a selection of accepted parameter sets chosen for their extreme parameter values, see Materials and Methods) was selected that has the minimal value for V_EGP_ (the cost function value calculated for (only) the five EGP data points in Fig 3A and 3C). The model outputs closely resemble the experimental data in both absolute value and relative response to different conditions. The model demonstrates that inhibition of the endogenous glucose production (EGP) by insulin is progressively reduced when the concentration of NEFA is increased during the different clamp conditions (Fig 3A and 3C), and that the inhibitory effect on insulin-dependent glucose uptake (i.e. insulin resistance) is dependent on the concentration of NEFA (Fig 3B and 3D). The model also reproduces the inability of NEFA to inhibit glucose uptake when the concentration of insulin is not elevated (Fig 3D). It should be noted that the original Dalla Man model [41] is unable to accurately simulate glucose uptake or EGP in the presence of NEFA (S1 Fig).

The new model is thus able to reproduce the clamp-datasets well. Next, it was investigated how uncertainty in the data propagates in the model outcome. In Fig 4, the model output for all parameter sets in S_sel_ is shown. In particular, for some selected sets of parameters EGP is completely suppressed in the clamp studies with basal insulin concentrations and hyperglycemia (dark green in Fig 4C). In other words, some of the parameter sets display good over-all agreement with data, even though they fail to reproduce the EGP data in Fig 3C. As only a few data points of EGP are included in the total cost function for model calibration, the full collection of accepted parameter values also contains parameter sets that yield unphysiological results for EGP. Since a correct description of the EGP is important for the physiological relevance of the model we trace how variability in EGP correlates with other model outcomes. Hereto the color scheme introduced in Fig 4 is repeated in the simulations shown in Figs 5, 6 and 8. The shade of green therefore denotes how well a particular parameter set fits the EGP data, where lighter green indicates a better fit to the EGP data.

### Simulation of dynamic response to oral challenges

Simulations of the model for postprandial dynamics following oral challenges demonstrate that the model is able to reproduce the dynamic response seen following the ingestion of glucose (OGTT, Fig 5A) and cream (OFTT, Fig 5B). In Fig 5, accepted parameter sets S_sel_ (green) are compared with parameter sets S_ext_ (grey). S_ext_ is a larger collection of parameter sets than S_sel_ and includes solutions with unphysiologically low values of the minimal stomach emptying parameter *k*_*ra3*_ (Materials and Methods).

In response to the glucose challenge (Fig 5A), plasma TG concentration changes little, while the concentration of glucose displays a transient peak at approximately 50 minutes followed by a non-monotonic decrease up to an undershoot at 240 minutes (Fig 5A, second panel). The concentration of plasma NEFA, meanwhile, shows an initial decrease in response to the postprandial insulin peak, followed by a post-absorptive overshoot (Fig 5A, forth panel). This behavior is reproduced well by the model. Between 120 and 240 minutes, no data are available, and this is reflected in a large variation in the dynamics within *S*_*ext*_ (grey), including some responses that display a large second peak of glucose appearance.

The OFTT dynamics are characterized by a lack of glucose or insulin response and a slow, transient increase in plasma TG concentration, visible in both the model and data (Fig 5B). A small decline in the concentration of NEFA is followed by a late overshoot, which is more moderate than in the response to the OGTT. The model is able to capture these responses well. For all the different parameter sets in *S*_*sel*_, integration of the simulated glucose ingestion flux results in a total glucose ingestion that entails far less than one gram of glucose (S3 File). As a negligible amount of glucose is ingested, the glucose ingestion parameters hardly contribute to the model response during an OFTT simulation and therefore are unidentifiable. Therefore, when selecting the parameter sets for *S*_sel,_ these parameters are not included in the selection of the most extreme parameter sets.

### The NEFA kinetic parameters are compatible with independent data

Following model calibration, the model was used to predict the response to an independent clamp experiment. The independent data were obtained from a study by Campbell *et al*. [53] and describes the response of NEFA metabolism to a series of euglycemic hyperinsulinemic clamps in which the insulin infusion rate is stepwise increased every two hours (Fig 6).

In the simulation, the initial concentration of NEFA was fixed to the basal NEFA concentration as reported in Campbell *et al*. [53], and the concentrations of glucose and insulin were fixed to the reported values per cascade step, while TG was fixed at 1 mM because data were not available (Fig 6A and 6B). The final insulin concentration of 13450 pM in [53] was not simulated, as the insulin concentration during this step is far outside the normal physiological ranges. The model correctly predicts the experimentally measured decline in the concentration of NEFA in response to step-wise increased concentrations of insulin (Fig 6C), although the third data point is underestimated for some parameter sets.

### Model simulations predict that glucose homeostasis is maintained during perturbations of NEFA metabolism

To better understand the behavior of the model, we performed a series of simulations in which NEFA metabolism was perturbed and compared the changes in the area under the curve (AUC) of glucose, insulin, and NEFA during the OGTT simulations (Fig 7). In the first series of perturbations, we simulated an increased initial concentration of NEFA (as demonstrated in Fig 7C), and in the second series, we reduced postprandial inhibition of lipolysis by insulin (as demonstrated in Fig 7D).

Both perturbations can be seen to clearly affect the simulated NEFA concentration–i.e. the AUC is increased (Fig 7E and 7F). In contrast, the AUC of the glucose response remains close to the unperturbed AUC (Fig 7G and 7H). However, the AUC of insulin is increased (Fig 7I and 7J), and this indicates that the maintained glucose homeostasis is the result of insulin control.

### Model analysis reveals that NEFA may mediate up to 45% of the postprandial glucose control

Finally, we used the model to determine the relative contribution of regulation by NEFA in the control of *EGP* and *U*_*id*_ (Fig 8). *U*_*id*_ is controlled by insulin (activation) and NEFA (inhibition), as described in Materials and Methods. *EGP* is a summation of inhibition by glucose itself (glucose effectiveness), inhibition by insulin and decrease of this inhibition by NEFA. However, because NEFA concentrations decrease rapidly in response to the OGTT, the effective regulation of NEFA during this period is to inhibit *EGP* and stimulate *U*_*id*_. In the OGTT simulations, we determined the maximal contribution of the NEFA regulation terms in relation to the full regulation of *EGP* and *U*_*id*_, for each of the parameter sets in S_sel_. For every parameter set, we plotted the (relative) contribution of the NEFA dependent regulation term to the total regulation at the time point where the NEFA-regulation term is maximal (Fig 8). A more detailed explanation of the calculations for Fig 8 is provided in the S4 File.

The results show that in the model, the regulation of *U*_*id*_ by NEFA (Fig 8A) is consistent in all parameter sets with a maximal value at approximately 100 min and is a contributor (30–45%) to *U*_*id*_ during the late postprandial period. The regulation of EGP by NEFA (Fig 8B) is more variable between the parameter sets. Nevertheless, the coloring shows that the parameter sets in which the contribution is low correspond to a poor value of *V*_*EGP*,_ i.e. to a poor agreement with the EGP clamp data in Fig 4A and 4C.

## Discussion

We have developed a model of postprandial systemic metabolism of glucose, insulin, and NEFA in humans, in order to describe and quantify the dynamic interplay between systemic glucose and NEFA homeostasis following the ingestion of an oral challenge. The model describes the calibration data and exhibits robust insulin-mediated compensation for perturbations of NEFA metabolism. By analyzing multiple parameter sets that reasonably describe the data, we have obtained a collection of responses that provides insight into the uncertainty of parameters and predictions. The value of the model is demonstrated in the analysis of postprandial metabolism of glucose, in which the postprandial decrease of the concentration of NEFA is shown to have a substantial contribution to the regulation of *EGP* and insulin-dependent glucose uptake. Specifically, in the later stages of the response to an oral glucose challenge (100–150 minutes after the ingestion of glucose), the model predicts that the NEFA-mediated signal has a contribution of between 30 and 45% to insulin-dependent glucose uptake, and a similar contribution to EGP. The model thus provides an important step towards untangling the postprandial dynamics of the interplay of glucose and lipid metabolism.

Our study integrates NEFA kinetics in a systemic model of glucose metabolism [41], and extends and adapts the equations describing NEFA kinetics in the model developed by Roy and Parker [49] in several directions. First, the model in [49] was stripped of the direct glucose-to-NEFA regulation, for which it lacked *in vivo* data. Next, the equations were extended to capture two characteristics of the data: (i) the absence of a steady state concentration of NEFA under fasting conditions, and (ii) the additional influx of NEFA, as seen following a lipid-rich meal, from adipose tissue spillover. The first extension (i) is of importance to differentiate the postprandial changes in concentrations of NEFA from the natural variation of NEFA concentrations seen even if no oral challenge is ingested [54]. Additionally, the first extension is necessary to reproduce the initial phase of the postprandial response [50]. The second extension (ii) is of importance because the spillover of NEFA from hydrolysis of circulating TG has a large contribution to the post-absorptive overshoot in the NEFA concentration [42,55]. Therefore, as the NEFA kinetic model [49] did not include postprandial spillover, physiological equations to describe this were included, as derived in [42].

The method and model proposed herein provide an *in vivo* estimate of the postprandial glucose-NEFA-insulin interactions at a systemic level, and provide information not accessible without a dynamic model. In a different approach presented in [56], glucose-NEFA interactions were quantified based on random perturbations in the fasting state in dogs, using Principal Dynamic Modes. Such an approach has several limitations in comparison to our approach, as the method neither includes the postprandial response, nor provides a detailed-, flux-based overview of the glucose-insulin-NEFA regulatory system. It is also possible to probe the NEFA contribution in the postprandial responses experimentally, e.g. by changing the initial NEFA concentration [57,58]. However, such perturbations are difficult to interpret in practice, since the NEFA concentration is a dynamic part of the system and thus changes continually; systemic responses to such perturbations are complex, involving several feedbacks and direct and indirect effects. In contrast, with our model we can study the contribution of NEFA without perturbing the response. Instead, we incorporate the effect of NEFA on glucose production and clearance as measured in clamp studies in a model that describes the postprandial situation. In healthy subjects, the glucose-insulin system maintains glucose homeostasis in spite of external influences, and our model reflects this tight control. The robustness of glucose homeostasis to perturbations in the concentration of NEFA has been retained in the new model (Fig 7). The effect of perturbations in NEFA metabolism on glucose metabolism presents itself mainly through increased insulin concentrations.

Some limitations of the approach must also be considered. First of all, limitations stem from combining several models and several datasets from literature. This implies that we assume all data represents the mean of the same population (of lean, healthy subjects) under comparable conditions. This may not, in all cases, be applicable, and this may hinder simultaneous agreement of the model with all data. Secondly, while the included datasets capture the key characteristics of the interaction we investigate, they are limited in their detail of NEFA metabolism. Therefore, only information on the net value of the NEFA fluxes is included in the dataset, and therefore only the net fluxes are described in the model. In other words, the NEFA kinetics are represented well by the model, but these are calculated from net fluxes, and does not distinguish between e.g. an increased uptake and a lowered release of NEFA. Absolute or tissue-specific NEFA fluxes remain an area for future development. In the literature, postprandial adipose [19] and hepatic [14] lipid fluxes have been quantified. These studies provide a scope for future model testing, validation, and extension.

The model presented here combines strengths of several included previously existing models, but simultaneously inherits limitations from each of these sources. In particular, our model contains a large number of remote insulin compartments that each represent a different phenomenological delayed signal, the kinetics of which are inherently difficult to identify as there are no direct measurements of these delays. A second model limitation is that we have not included a direct stimulatory effect of NEFA on the release of insulin from the β-cells [59]. In the model, the effect of NEFA on insulin is instead achieved as a result of the ability of NEFA to increase the circulating concentration of glucose, which in turn increases insulin release to the circulation. Finally, the model requires the input of TG concentration for a calculation of NEFA influx due to spillover. These TG measurements are not always available, and for this reason the concentration of TG was fixed to a constant value in the simulation of the independent dataset (Fig 6).

In the future, continued development of the model will allow further analyses of the interplay of glucose–NEFA. Model extensions could include data describing mixed meals or detailed NEFA fluxes. A model that has been extended with organ-specific fluxes of NEFA could be used to connect more detailed models of tissue and organ metabolism. Such a model could translate between drug simulations of intracellular signaling and metabolism to the whole-body level, as previously done for the dynamics of glucose and insulin [44]. Mathematical models have been shown to be useful in elucidating disease development [60,61] and therefore another interesting avenue for future model application is in the investigation of the development of metabolic disease–particularly Type 2 Diabetes (T2D). The defining feature of T2D is a loss of the control of blood glucose concentrations, which is due to insulin resistance and insufficient insulin secretion. However, the detailed mechanism underlying T2D [62–64] is still not fully known. Dysregulation of glucose levels in T2D is regularly accompanied by a corresponding dysregulation of lipid metabolism: elevations of circulating glucose levels are accompanied by elevations of the levels of NEFA and TG [65,66]. The herein proposed model-based estimation of the interplay between glucose and lipid metabolism is useful to gain better insight in the changes in this interplay in the postprandial period and can contribute to a better understanding of development of T2D.

## Supporting Information

S1 Fig Simulation of clamp datasets with the Dalla Man model. Data of clamp datasets (as Figs 3 and 4) with Dalla Man model simulations superimposed.
(EPS)

S1 File Simulations details. Description of the simulation corresponding to each dataset, as well as details of model optimization.
(PDF)

S2 File Model equations. Model equations and parameter values for the Glucose-NEFA model and the Dalla Man model.
(PDF)

S3 File Simulation files. Zip file containing all Matlab m-files to generate figures. The zip-file includes a README file, containing an overview of the simulation files and basic instructions.
(ZIP)

S4 File Model analysis. Equations and implementation of (1) analysis of the relative control of NEFA in postprandial glucose metabolism and (2) analysis of model response to NEFA metabolism perturbation.
(PDF)
