# Supplementary material for: Model-Based Quantification of the Systemic Interplay between Glucose and Fatty Acids in the Postprandial State
Source: PLoS One. 2015 Sep 10;10(9):e0135665. doi: 10.1371/journal.pone.0135665 (PMC4565650; doi:10.1371/journal.pone.0135665)
Supplement: S1 File — Description of the simulation corresponding to each dataset, as well as details of model optimization. (PDF) [file pone.0135665.s002.pdf]

S1. Simulations details.

Supplemental Data (file S1) for:

Model-based quantification of the systemic interplay between glucose and fatty acids in the postprandial state

Fianne Sips, Elin Nyman, Martin Adiels, Peter Hilbers, Peter Strålfors, Natal van Riel, Gunnar Cedersund

This supplementary file includes an overview of included datasets (Table 1), and the details of the implementation of D_{CLAMP1} (Table 2), D_{CLAMP2} (Table 3, Figure 1), D_{MEAL} (Table 4) and D_{ID} (Table 5), as well as an overview of estimated parameters and bounds (Table 6). Finally, it describes in detail the procedure of selecting S_{sel} via a bound on k_{ra3}.

Table 1. Overview of data. *Symbols in the Table denote the following: * The scaling of the insulin concentrations in D_{MEAL} was corrected in order to comply with the reported basal value in* **[1]**. *** TG is used as model input via polynomial fit or interpolation. *** D_{ID} is not part of the calibration dataset.*

Measurement	Glucose	Insulin	NEFA	TG	EGP	GU
Unit	mg/dL	$pmol/L$	$\mu mol/L$	$\mu mol/L$	$mg/kg/min$	$mg/kg/min$
D_{MEAL}	10 +10	6 + 6*	10 + 10	12 + 12**	-	-
D_{CLAMP1}	-	-	-	-	1 + 1 + 1	1 + 1 + 1
D_{CLAMP2}	-	-	-	-	0 + 0 + 2	2 + 2 + 2
D_{ID} ***	-	-	3	-	-	-

Table 2. Implementation of D_{CLAMP1}. *Symbols in the Table denote the following: * These concentrations are initialized at the basal value, and set to rise/fall to the fixed value within the first minute.*

Initial condition	*Unit*	*Time*	*Value*			*Notes*
			A	B	C	
G_b	mg/dL		85	85	85	
I_b	$pmol/L$		30	30	30	
$NEFA_b$	$\mu mol/L$		526	500	415	
EGP_b	$mg/kg/min$		2.36	2.36	2.36	Mean of 1,2,3
S_b	$pmol/kg/min$		1.55	1.55	1.55	Unmeasured, and thus fixed to the value in [2].
Fixed						
$G(t)$	mg/dl	*	85	85	85	$G_p(t) = G(t) \cdot v_g$
$I(t)$	$pmol/L$	*	420	420	420	$I_p(t) = I(t) \cdot v_i$
$NEFA(t)$	$\mu mol/L$	*	51	562	766	
$\dot{G}$	$mg/dL/min$	*	0	0	0	
$ins_{inf}(t)$	$pmol/kg/min$	*	6	6	6	

Table 3. Implementation of D_{CLAMP2}. *Symbols in the Table denote the following: * Fixed from 20 minutes (rising between 19.5-20.5 minutes), see Fig. 1*

Initial condition	*Unit*	*Time*	*Value*						*Notes*
			A-	A+	B-	B+	C-	C+	
G_b			89.0	89.0	83.6	80.9	81.8	77.1	
I_b			94.1	80.4	82.8	74.5	84	84	
$NEFA_b$			478	576	448	456	650	593	
EGP_b			-	-	-	-	1.9	1.8	
S_b			1.55	1.55	1.55	1.55	1.55	1.55	Unmeasured, and thus fixed to the value in [2].
Fixed									
$G(t)$		*	89	89	204	206	291	287	$G_p(t) = G(t) \cdot v_g$
$I(t)$		*	738	834	288	300	96	126	$I_p(t) = I(t) \cdot v_i$
$N(t)$		*	-	-	-	-	-	-	Interpolated, please see Figure D.1
$\dot{G}$		*	0	0	0	0	0	0	From 20 minutes.
$I_{inf}(t)$		*	*Not reported*	*Not reported*	*unused*	*unused*	0	0	Only used for determination of EGP, thus not implemented in A or B.

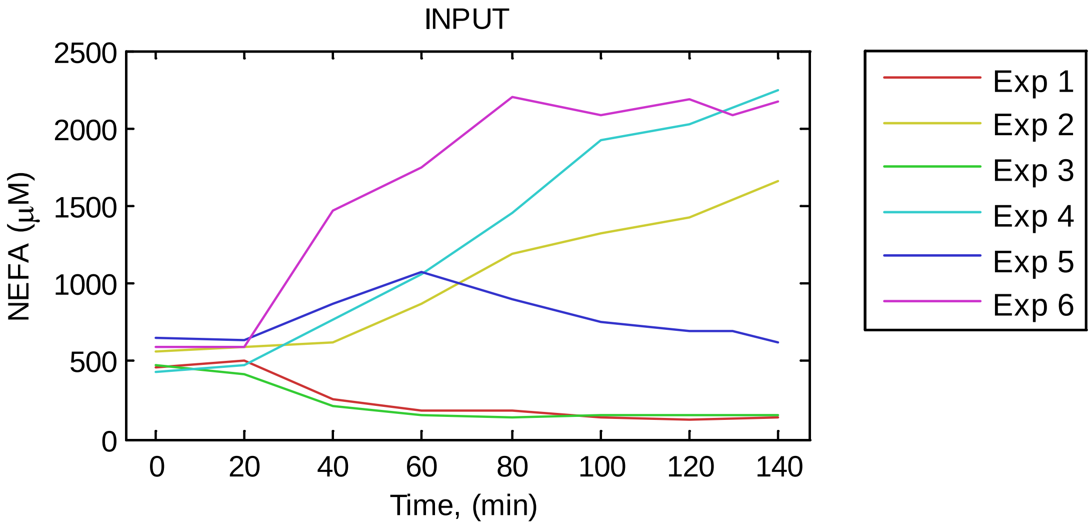

Figure 1: Fixed NEFA in the simulation of D_{CLAMP2}. Exp 1 is A-, Exp 2 is A+, Exp 3 is B-, Exp 4 is B+, Exp 5 is C-, Exp 6 is C+.

Table 4. Implementation of D_{MEAL}.

TG is calculated via $\boldsymbol{TG = TG1\ t^4 + TG2\ t^3 + TG3\ t^2 + TG4\ t + TG5}$*. The scaling of the insulin concentrations in D_{MEAL} was corrected to the in order to comply with the reported basal value in* **[1]**. *Conversion of glucose and insulin was performed according to* **[3]** *(0.05551 mmol/L / mg/dL) and* **[4]** *(6 pmol/L / μU/mL), respectively.*

Initial condition	***Unit***	***Value***		***Notes***
		OGTT	OFTT	
G_b	md/dL	78.2	80.8	Initial point of digitized data
I_b	$pmol/L$	50.5	50.5	Initial point of digitized data
$NEFA_b$	$\mu mol/L$	410	437	Initial point of digitized data
EGP_b	$mg/kg/min$	1.9	1.9	Unmeasured, and thus fixed to the value in [2].
S_b	$pmol/kg/min$	1.55	1.55	Unmeasured, and thus fixed to the value in [2].
$TG1$	$\frac{\mu mol/L}{min^4}$	$1.1307\mathrm{e}-07$	$7.9243\mathrm{e}-07$	
$TG2$	$\frac{\mu mol/L}{min^3}$	$-4.4882\mathrm{e}-05$	$-6.2738\mathrm{e}-04$	
$TG3$	$\frac{\mu mol/L}{min^2}$	-0.0010	0.1334	
$TG4$	$\frac{\mu mol/L}{min}$	0.9596	-3.9547	
$TG5$	$\mu mol/L$	1091.9	1115.5	

Table 5. Implementation of D_{ID}

Variable	*Unit*	*Value*	*Notes*
t	min	$[Basal \quad 0-120 \quad 120-240 \quad 240-360]$	
G	mg/dL	$[88.3 \quad 84.7 \quad 91.9 \quad 95.5]$	
I	$pmol/L$	$[54 \quad 84 \quad 192 \quad 468]$	
EGP_b	$mg/kg/min$	1.9	Unmeasured, and thus fixed to the value in [2].
S_b	$pmol/kg/min$	1.55	Unmeasured, and thus fixed to the value in [2].
$TG1$	$\frac{\mu mol/L}{min^4}$	**0**	
$TG2$	$\frac{\mu mol/L}{min^3}$	0	
$TG3$	$\frac{\mu mol/L}{min^2}$	0	
$TG4$	$\frac{\mu mol/L}{min}$	0	
$TG5$	$\mu mol/L$	1000	Constant TG

Table 6. Model parameters and parameter boundaries. *Symbols in the Table denote the following: (1) If the parameter is not included in the full calibration dataset, and thus only influences a subset of the simulations, the relevant subsets of data are noted here. (2) Parameter lower and upper bounds, as set in the optimization procedure. An entry of (-) indicates –* $\boldsymbol{Inf}$ *(lower bound) and* $\boldsymbol{Inf}$ *(upper bound). (3) Note that parameter* $\boldsymbol{k_{egp2}}$ *– direct influence of glucose on EGP – is fixed, for two reasons: First, the majority of EGP clamp data is obtained from euglycemic conditions (in which glucose is fixed at basal) and thus does not contain additional information on glucose regulation of EGP. Secondly, the added term of NEFA-mediated regulation is a "slow" term, and thus is expected to be closely related to the* $\boldsymbol{k_{egp3}}$ *term – which must be re-estimated following introduction of* $\boldsymbol{k_{egp5}}$*. (4) Note that parameter* $\boldsymbol{k_{ra4}}$ *is fixed, as the previously determined values were found to be consistent and meal-independent [***[2,5]***].*

Parameter	Datasets[1]	Unit	Low bound[2]	High bound[2]	Explanation
v_g		dL/kg	1.49	3.0	Because the distribution volume of glucose is not constant, but rather relates to the dynamics of the situation in addition to the model [6], we expect that the volume of distribution necessary to describe clamp experiments in addition to meal experiments may differ from the previously determined value. A wide range of values for the distribution volume following clamp experiments can be found in the literature [6],[7]. We allow v_g to vary from a value of 1.49 ([2], T2D) to a value of 3, such that the total distribution volume (of $G_p + G_t$) may reach a value of ~0.49 L/kg, as reported in [7] for a model describing clamp responses.
p_A		min^{-1}	0	–	
p_B		$\frac{\mu mol}{min \cdot pmol}$	0	–	

p_C		$\frac{\mu mol}{min \cdot L}$	–	–	
A_{spill}		%	–	–	The final values of *spill* (Eq. GN4g) should be between 0 and 1.
B_{spill}		%	–	–	The final values of *spill* (Eq. GN4g) should be between 0 and 1.
k_{egp3}[3]		$\frac{mg/kg/min}{pmol/L}$	0	–	
k_{egp4}		$\frac{mg/kg/min}{pmol/kg}$	0	–	
k_{egp5}		$\frac{mg/kg/min}{\mu mol/L}$	0	–	
k_{uid3}		$\frac{mg/kg/\min \cdot \mu mol/L}{pmol/L}$	0	–	
$K_{m,uid}$		mg/kg	0	–	
θ_{OGTT}	$D_{MEAL,\ OGTT}$	–	0.8	1	For the mixed meal reported in [2], θ is reported to be 0.9. We assume the value of θ may be meal dependent and thus allow a small range of values in which θ is estimated. The lower bound for this range is chosen based on previously published values ([8]; $\theta = 0.86$ and [9]; $\theta = 0.8$) as well as calculation of θ for several datasets of the OGTT rate of appearance ([10]/[11], [12], [13]). The upper bound is 1 such that glucose appearance cannot exceed glucose intake.
$d\delta_{OGTT}$	$D_{MEAL,\ OGTT}$	–	0	1	Due to the shape of k_{ra2} (eq. DM3e) it holds that $\delta < 1$. For δ, it also must hold that $\delta > \varepsilon$. To enforce this, we define $\delta = \varepsilon + d\delta$ and estimate $d\delta$. For $d\delta$ it also holds that the value must be between 0 and 1.
ε_{OGTT}	$D_{MEAL,\ OGTT}$	–	0	1	Due to the shape of k_{ra2} (eq. DM3e) it holds that $\varepsilon < 1$
$k_{ra3,OGTT}$[4]	$D_{MEAL,\ OGTT}$	min^{-1}	0	0.0558	Due to the shape of k_{ra2} (eq. DM3e) it holds that $k_{ra3} < k_{ra4}$
$k_{ra1,OGTT}$	$D_{MEAL,\ OGTT}$	min^{-1}	0	–	
θ_{OFTT}	$D_{MEAL,\ OFTT}$	–	0	1	In the OFTT, only a small amount of complex carbohydrates is present. We allow θ to vary between 0 and 1 and thus do not enforce that all carbohydrates appear in plasma.
$d\delta_{OFTT}$	$D_{MEAL,\ OFTT}$	–	0	1	See $d\delta_{OGTT}$.
ε_{OFTT}	$D_{MEAL,\ OFTT}$	–	0	1	See ε_{OGTT}.
$k_{ra3,OFTT}$	$D_{MEAL,\ OFTT}$	min^{-1}	0	0.0558	See $k_{ra3,OGTT}$.
$k_{ra1,OFTT}$	$D_{MEAL,\ OFTT}$	min^{-1}	0	–	

Selection of S_{sel}

To select the parameter sets of S_{sel}, we impose a boundary on parameter k_{ra3}. This boundary is based on the values previously determined for this parameter in the publications [5,10] (in which the parameter is referred to as k_{min}). In [5], a comparison of meal and OGTT rate of appearance yielded the observation that this value is larger for OGTT (average value 0.006 ± 0.001 for meal, 0.013 ± 0.002 for OGTT). The 95 % CIs yield a boundary between these values of between 0.008 and 0.009; a value of 0.009 was therefore chosen as the minimal value for OGTT absorption.

References

1. Robertson MD, Henderson RA, Vist GE, Rumsey RDE. Extended effects of evening meal carbohydrate-to-fat ratio on fasting and postprandial substrate metabolism. Am J Clin Nutr. 2002;75: 505–510.

2. Dalla Man C, Rizza RA, Cobelli C. Meal simulation model of the glucose-insulin system. IEEE Trans Biomed Eng. 2007;54: 1740–1749. doi:10.1109/TBME.2007.893506

3. YOUNG DS. Implementation of SI Units for Clinical Laboratory DataStyle Specifications and Conversion Tables. Ann Intern Med. 1987;106: 114–129. doi:10.7326/0003-4819-106-1-114

4. Vølund A, Brange J, Drejer K, Jensen I, Markussen J, Ribel U, et al. In vitro and in vivo potency of insulin analogues designed for clinical use. Diabet Med J Br Diabet Assoc. 1991;8: 839–847.

5. Dalla Man C, Camilleri M, Cobelli C. A system model of oral glucose absorption: validation on gold standard data. IEEE Trans Biomed Eng. 2006;53: 2472–2478. doi:10.1109/TBME.2006.883792

6. Wolfe RR, Chinkes DL. Isotope Tracers in Metabolic Research: Principles and Practice of Kinetic Analysis. John Wiley & Sons; 2005.

7. Picchini U, Gaetano AD, Panunzi S, Ditlevsen S, Mingrone G. A mathematical model of the euglycemic hyperinsulinemic clamp. Theor Biol Med Model. 2005;2: 44. doi:10.1186/1742-4682-2-44

8. Dalla Man C, Caumo A, Cobelli C. The oral glucose minimal model: estimation of insulin sensitivity from a meal test. IEEE Trans Biomed Eng. 2002;49: 419–429. doi:10.1109/10.995680

9. Caumo A, Bergman RN, Cobelli C. Insulin Sensitivity from Meal Tolerance Tests in Normal Subjects: A Minimal Model Index. J Clin Endocrinol Metab. 2000;85: 4396–4402. doi:10.1210/jcem.85.11.6982

10. Dalla Man C, Caumo A, Cobelli C. The oral glucose minimal model: estimation of insulin sensitivity from a meal test. IEEE Trans Biomed Eng. 2002;49: 419–429. doi:10.1109/10.995680

11. Dalla Man C, Yarasheski KE, Caumo A, Robertson H, Toffolo G, Polonsky KS, et al. Insulin sensitivity by oral glucose minimal models: validation against clamp. Am J Physiol Endocrinol Metab. 2005;289: E954–959. doi:10.1152/ajpendo.00076.2005

12. Mitrakou A, Kelley D, Mokan M, Veneman T, Pangburn T, Reilly J, et al. Role of reduced suppression of glucose production and diminished early insulin release in impaired glucose tolerance. N Engl J Med. 1992;326: 22–29. doi:10.1056/NEJM199201023260104

13. Rose AJ, Howlett K, King DS, Hargreaves M. Effect of prior exercise on glucose metabolism in trained men. Am J Physiol Endocrinol Metab. 2001;281: E766–771.
