# Supplementary material for: Model-Based Quantification of the Systemic Interplay between Glucose and Fatty Acids in the Postprandial State
Source: PLoS One. 2015 Sep 10;10(9):e0135665. doi: 10.1371/journal.pone.0135665 (PMC4565650; doi:10.1371/journal.pone.0135665)
Supplement: S2 File — Model equations and parameter values for the Glucose-NEFA model and the Dalla Man model. (PDF) [file pone.0135665.s003.pdf]

S2. Model equations

Supplemental Data (file S2) for:

Model-based quantification of the systemic interplay between glucose and fatty acids in the postprandial state

Fianne Sips, Elin Nyman, Martin Adiels, Peter Hilbers, Peter Strålfors, Natal van Riel, Gunnar Cedersund

Glucose NEFA model equations

We note that all constants, variables, parameters and initial conditions are listed below (Tables 1-2).

The model builds upon the meal simulation model developed by Dalla Man *et al* (2007) [1], which will be referred to as M_{DM} in this document and is described in detail below. The equation describing plasma **glucose** content is largely derived from M_{DM} (equations DM1 – 6). In short, the plasma glucose concentration can be described by equation GN1b, which is derived from M_{DM} plasma glucose by replacing equations DM2a and DM2b with equations GN2a and GN2b, and replacing equation DM5f with equation GN3.

$$\frac{d\,G_p(t)}{d\,t} = EGP(t) + Ra(t) - U_{ii} - E(t) - trans_{tp}(t) + trans_{pt}(t), \qquad G_p(0) = G_{pb} \quad \text{(GN1a)}$$

$$G(t) = \frac{G_p(t)}{v_g}, \qquad G(0) = G_b = \frac{G_{pb}}{v_g} \quad \text{(GN1b)}$$

In the M_{GN} model, EGP is NEFA-dependent (GN2a).

$$EGP(t) = k_{egp1} - k_{egp2}\,G_p(t) - k_{egp3} I_{d2}(t) - k_{egp4}\left(I_{po}(t) + \frac{ins_{inf}(t)}{\gamma}\right) + k_{egp5}\,N_{d1}(t), \quad \text{(GN2a)}$$

Where $G_p(t)$is the plasma glucose content (mg/kg, Equation GN1a), $I_{d2}(t)$ is a delayed insulin compartment described below by equation DM2d, $I_{po}(t)$ is the portal vein insulin concentration described by equation DM10a, $N_{d1}(t)$is a delayed NEFA signal described by equation GN5 and $ins_{inf}(t)$ is the insulin infusion rate in $pmol/kg/min$. In this equation, the first term is a variable (k_{egp1}) chosen such that $EGP(0) = EGP_b$. This is achieved by defining k_{egp1} as shown in Equation GN2b.

$$k_{egp1} = EGP_b + k_{egp2}\, G_p(0) + k_{egp3} I_{d2}(0) + k_{egp4}\, I_{po}(0) - k_{egp5}\, N_{d1}(0) \qquad \text{(GN2b)}$$

Uid is also NEFA-dependent (equation GN3). Here, $V_{max,uid}$ is the maximal rate of glucose uptake, and thus replaces the $V_{max,uid}$ defined in Equation DM5F.

$$V_{max,uid}\left((I_{d3}(t), N_{d1}(t))\right) = k_{uid1} + k_{uid3} \frac{I_{d3}(t)}{N_{d1}(t)} \qquad \text{(GN3)}$$

In both cases, NEFA dependency is thus mediated by remote compartment N_{dl} (*vide infra*).

Plasma insulin is described by M_{DM} equations 7-11.

Plasma NEFA is described by equation GN4a, and consists of four main components (GN4b-e).

$$\frac{d\,NEFA}{dt} = -J_A - J_B + J_C + J_D \qquad NEFA(0) = NEFA_b \quad \text{(GN4a)}$$

$$J_A = p_A\, NEFA \qquad \text{(GN4b)}$$

$$J_B = p_B\,(I_{d7} - I_b)\;\; NEFA \qquad \text{(GN4c)}$$

$$J_C = p_C \qquad \text{(GN4d)}$$

$$J_D = \frac{spill \cdot J_{lpl}}{V_N} \qquad \text{(GN4e)}$$

Here, J_A represents the NEFA-dependent uptake of NEFA by liver and muscle [2], J_B represents the insulin-dependent decrease in adipose tissue lipolysis [2], J_C represents the constant fasting NEFA uptake, and J_D represents adipose tissue spillover of fatty acids derived from plasma triglycerides [3].

J_B depends on remote compartment I_{d7} [2].

$$\frac{d\,I_{d7}}{dt} = -p_D(I_{d7} - I_b) + p_E(I - I_b) \qquad I_{d7}(0) = I_b \quad \text{(GN 4f)}$$

Postprandial spillover is a major contributor to postprandial NEFA concentrations [4] and therefore must be included in the model. J_D is governed by an insulin-dependent spillover term *spill,* which is related to adipose tissue delay term I_{d6} (as the time characteristics of the insulin related decrease of adipose tissue spillover is reported to be on a similar time scale to postprandial LPL induction [5]).

$$spill = \frac{A_{spill} + B_{spill}\frac{I_b}{I_{d6}}}{100} \quad \text{(GN4g)}$$

J_{lpl}, meanwhile, is given by equations GN4h-4k, and derived from [3]

$$J_{lpl} = \frac{LPL_{max} \cdot TG}{K_{lpl} + TG} \cdot \frac{P \cdot I_{d6}}{K_p + I_{d6}} \quad \text{(GN4h)}$$

$$\frac{d\, I_{d4}}{d\, t} = \frac{3}{\tau_{lpl}} (I - I_{d4}) \qquad I_{d4}(0) = I_b \quad \text{(GN4i)}$$

$$\frac{d\, I_{d5}}{d\, t} = \frac{3}{\tau_{lpl}} (I_{d4} - I_{d5}) \qquad I_{d5}(0) = I_b \quad \text{(GN4j)}$$

$$\frac{d\, I_{d6}}{d\, t} = \frac{3}{\tau_{lpl}} (I_{d5} - I_{d6}) \qquad I_{d6}(0) = I_b \quad \text{(GN4k)}$$

Finally, the remote compartment $N_{d1}(t)$ – derived from [2] – is given by:

$$\frac{N_{d1}(t)}{dt} = p_H\,(NEFA - p_G) - p_F\,(N_{d1} - p_G) \qquad N_{d1}(0) = N_{d1b} \quad \text{(GN5)}$$

With $N_{d1b} = p_H \frac{NEFA_b - p_G}{p_F} + p_G$

Table 1 Constants and parameters of the M_{GN} model

Symbol	Unit	Value	Description and source
EGP_b	$mg/kg/min$	Fixed to healthy or – if available – from dataset.	Basal endogenous glucose production
k_{egp1}	$mg/kg/min$	Derived from basal state (equation GN2b).	Maximal endogenous glucose production
k_{egp2}	min^{-1}	0.0021	Parameter governing glucose feedback on EGP, [1]
k_{egp3}	$\frac{mg/kg/min}{pmol/L}$		Parameter for delayed insulin regulation of EGP, [1]
k_{egp4}	$\frac{mg/kg/min}{pmol/kg}$		Parameter governing insulin regulation of EGP, [1]
k_{egp5}	$\frac{mg/kg/min}{\mu mol/L}$		Parameter governing NEFA regulation of EGP
k_{uid3}	$\frac{mg/kg/\min\cdot\mu mol/L}{pmol/L}$		Insulin- and NEFA-dependent term of $Vmax$
V_N	L	3	Distribution volume of NEFA, [3]
p_A	min^{-1}		NEFA uptake by tissues, mass action term [6].
p_B	$\frac{\mu mol\ /\ L}{pmol/L\ \cdot min}$		NEFA uptake by tissues, insulin regulation term [6].
p_C	$\frac{\mu mol\ /\ L}{min}$		NEFA uptake, basal term.
p_D	min^{-1}	0.17	Parameter governing I_{d7} [2].
p_E	min^{-1}	$1 \cdot 10^{-5}$	Parameter governing I_{d7} [2].
p_F	min^{-1}	0.03	Parameter governing N_{d1} [2].
p_G	$\mu mol/\ L$	380	Parameter governing N_{d1} [2].
p_H	min^{-1}	0.02	Parameter governing N_{d1} [2].
A_{spill}	–		Constant spillover term.
B_{spill}	–		Insulin dependent spillover term.
LPL_{max}	$\mu mol/\ min$	350	Maximal rate of LPL FA release, [3]
K_{lpl}	$\mu mol/L$	3000	Parameter of LPL action, [3]
K_p	$pmol/L$	200	Parameter of LPL activation, [3]
τ_{lpl}	min	240	Delay of remote insulin compartments ($I_{d4} - I_{d6}$), [3]

Table 2 Variables, initial conditions, inputs of the M_{GN} model

Symbol	Unit	Description	Equation / value
**Variables**			
P	$-$	Parameter of LPL activation, [3]	$\frac{K_p + I_b}{I_b}$
**State variables**			
G_p	$mg/\,kg$	Plasma glucose content	Equation GN1a
G_t	$mg/\,kg$	Tissue glucose content	Equation DM 5a
Q_{sto1}	mg	1st stomach compartment	Equation DM 3b
Q_{sto2}	mg	2nd stomach compartment	Equation DM 3c
Q_{gut}	mg	Gut compartment	Equation DM 3d
I_{d1}	$pmol/L$	Insulin delay (EGP)	Equation DM 2c
I_{d2}	$pmol/L$	Insulin delay (EGP, following I_{d1})	Equation DM 2d
I_{d3}	$pmol/L$	Insulin delay (U_{id})	Equation DM 5g
I_p	$pmol/kg$	Plasma insulin content	Equation DM 8a
I_l	$pmol/kg$	Hepatic insulin content	Equation DM 7a
Ipo	$pmol/kg$	Portal vein insulin content	Equation DM 10a
Y	$pmol/kg/min$	Glucose delayed control of insulin secretion	Equation DM 11
I_{d4}	$pmol/L$	Insulin delay (LPL)	Equation GN4i
I_{d5}	$pmol/L$	Insulin delay (LPL)	Equation GN4j
I_{d6}	$pmol/L$	Insulin delay (LPL)	Equation GN4k
$NEFA$	$\mu mol/L$	Plasma NEFA	Equation GN4a
N_{d1}	$\mu mol/L$	Remote compartment for plasma NEFA	Equation GN5
I_{d7}	$pmol/L$	Insulin delay (lipolysis)	Equation GN4f

Table 2 (continued)

**Initial conditions**			
I_{d4}	$pmol/L$	Equation GN4i	I_b
I_{d5}	$pmol/L$	Equation GN4j	I_b
I_{d6}	$pmol/L$	Equation GN4k	I_b
$NEFA$	$\mu mol/L$	Equation GN4a	$NEFA(0)$
N_{d1}	$\mu mol/L$	Equation GN5	$N_{d1b} = p_H \frac{NEFA_b - p_G}{p_F} + p_G$
I_{d7}	$pmol/L$	Equation GN4f	I_b
**Inputs**			
ins_{inf}	$pmol/kg/min$	Insulin infusion rate.	Default: 0

M_{DM} model equations

The M_{DM} model was developed by Dalla Man *et al* in [1] and [7]. Here, we provide the equations of the model, which has been adapted to accommodate glucose or insulin infusion. We note that the model is simulated as a whole, and not (as in [1]) in four separate unit process models.

The **plasma glucose** content $G_p(t)$ (mg/kg) is modelled as the sum of several sources of glucose (endogenous glucose production, $EGP(t)$; meal rate of appearance, $Ra(t)$; as well as several processes that remove glucose from circulation (insulin-independent glucose uptake, U_{ii}; insulin-dependent glucose excretion, U_{id}, which is modelled via a tissue glucose compartment; and renal excretion of glucose $E(t)$). The equation governing plasma glucose is given in Equation DM1a.

$$\frac{d\,G_p(t)}{d\,t} = EGP(t) + Ra(t) - U_{ii} - E(t) - trans_{tp}(t) + trans_{pt}(t), \qquad G_p(0) = G_{pb} \qquad \text{(DM1a)}$$

In this equation the right-hand side of the differential equation is composed of the six glucose fluxes ($EGP(t)$, $Ra(t)$, U_{ii}, $E(t)$, $trans_{tp}(t)$, and $trans_{pt}(t)$, all in $mg/kg/min$) that have been described in [1] and [7] and will be expanded on below.

The plasma glucose concentration can be calculated by dividing the plasma glucose content by the distribution volume of glucose (v_g, in dL/kg, see Table 3) as in Equation DM1b.

$$G(t) = \frac{G_p(t)}{v_g}, \qquad G(0) = G_b = \frac{G_{pb}}{v_g} \qquad \text{(DM1b)}$$

The **endogenous glucose production** ($EGP(t)$, $(mg/kg/min)$) represents the systemic glucose production, which originates from the liver and, to a lesser extent, the kidneys. It is regulated by three terms; (1) the plasma glucose concentration itself, (2) a (delayed) insulin signal and (3) insulin appearance (which has thus been expanded to include insulin infusion in clamps - $ins_{inf}(t)$). The endogenous glucose production is given in Equations DM2a-d.

$$EGP(t) = k_{egp1} - k_{egp2}\,G_p(t) - k_{egp3}I_{d2}(t) - k_{egp4}\left(I_{po}(t) + \frac{ins_{inf}(t)}{\gamma}\right), \qquad \text{(DM2a)}$$

In this equation, the first term is a variable (k_{egp1}) defining the basal production such that $EGP(0) = EGP_b$. This is achieved by defining k_{egp1} as shown in Equation DM2b.

$$k_{egp1} = EGP_b + k_{egp2}\,G_p(0) + k_{egp3}I_{d2}(0) + k_{egp4}\,I_{po}(0) \qquad \text{(DM2b)}$$

The second term is the direct feedback of the plasma glucose content and the insulin signal featured in the third term is the result of the two-step delay described by Equations DM2c and d.

$$\frac{d\,I_{d1}(t)}{dt} = -k_{d12}(I_{d1}(t) - I(t)), \qquad I_{d1}(0) = I_b \qquad \text{(DM2c)}$$

$$\frac{d\,I_{d2}(t)}{dt} = -k_{d12}(I_{d2}(t) - I_{d1}(t)), \qquad I_{d2}(0) = I_b \qquad \text{(DM2d)}$$

The fourth term, finally, depends on the portal vein insulin concentration which will be described in equation DM10a. k_{egp2}, k_{egp3} and k_{egp4} are parameters (Table 3).

The **rate of appearance** $Ra(t)$ $(mg/kg/min)$ is the rate at which orally ingested glucose and carbohydrates appear as glucose in circulation. The model is described in [7] and is given by Equations DM3a-h.

$$Ra(t) = \frac{\theta\ k_{ra1}\ Q_{gut}(t)}{BW} \qquad \text{(DM 3a)}$$

Equation DM3a relates the rate of appearance directly to the glucose mass in the gut ($Q_{gut}(t)$) and modulated by ϑ, k_{ra1} and BW (Table 3). The gut glucose content is the third of a three compartment digestive tract system, which describes the introduction of glucose to the (first) stomach glucose compartment ($Q_{sto1}(t)$, Equation DM3b), the turnover to a second stomach glucose compartment ($Q_{sto2}(t)$, Equation DM3c), and finally the emptying of the stomach into the gut compartment (k_{ra2}, Equation DM3d). Stomach emptying is dependent on stomach glucose mass and is described by Equations DM3e-h.

$$\frac{d\ Q_{sto1}(t)}{d\ t} = D\ d(t - t_{meal}) - k_{ra5}\ Q_{sto1}(t)\ , \qquad Q_{sto1}(0) = 0 \quad \text{(DM3b)}$$

The input of glucose is thus modelled via a Dirac function ($d(t - \tau)$) at time t_{meal}. Note that if $t_{meal} = 0$, then the initial value is not 0, but rather D. Transport to $Q_{sto2}(t)$ depends on k_{ra5}(Table C.1).

$$\frac{d\ Q_{sto2}\ (t)}{d\ t} = k_{ra5}\ Q_{sto1}(t) - k_{ra2}(Q_{sto}(t)) \cdot Q_{sto2}(t) \qquad Q_{sto2}(0) = 0 \quad \text{(DM3c)}$$

The equation for $Q_{sto2}\ (t)$ consists of the glucose exiting $Q_{sto1}\ (t)$, and the term describing stomach emptying (3e-h).

$$\frac{d\ Q_{gut}(t)}{d\ t} = -k_{ra1}\ Q_{gut}(t) + k_{ra2}(Q_{sto}(t)) \cdot Q_{sto2}(t) \qquad Q_{gut}(0) = 0 \quad \text{(DM3d)}$$

Finally, the emptying of Q_{gut} is modelled with mass action kinetics.

$$k_{ra2}\ (Q_{sto}) = k_{ra3} + \left(\frac{k_{ra4} - k_{ra3}}{2}\right) \{\tanh[\zeta(\ Q_{sto}(t) - \delta D)] - \tanh[\eta(\ Q_{sto}(t) - \varepsilon D)] + 2\} \quad \text{(DM3e)}$$

$$\zeta = \frac{5}{2\ D\ (1-\delta)} \qquad \text{(DM3f)}$$

$$\eta = \frac{5}{2\ D\ \varepsilon} \qquad \text{(DM3g)}$$

$$Q_{sto}(t) = Q_{sto1}(t) + Q_{sto2}(t) \qquad \text{(DM3h)}$$

Glucose uptake is the modelled as the sum of insulin-independent glucose uptake (e.g. for brain, erythrocytes, etc.) and insulin-dependent glucose uptake (e.g. muscle, adipose tissue, etc). Insulin-independent glucose uptake is modelled as a constant flux out of the plasma compartment (Equation DM4).

$$U_{ii} = 1 \qquad \text{(DM4)}$$

Insulin-dependent glucose uptake, meanwhile, takes place in tissue compartment G_t. The glucose content in G_t (Equation DM5a) is the sum of transport from plasma to tissue ($trans_{tp}$, Equation DM5b), reciprocal transport from tissue to plasma ($trans_{pt}$, Equation DM5c) and insulin-dependent glucose uptake (U_{id}, Equation DM5d).

$$\frac{d\ G_t(t)}{d\ t} = trans_{tp}\ (t) - trans_{pt}(t) - U_{id}(t)\ , \qquad G_t(0) = G_{tb} \quad \text{(DM5a)}$$

$$trans_{tp}\ (t) = k_{tr1}\ G_p(t) \qquad \text{(DM5b)}$$

$$trans_{tp}\ (t) = k_{tr2}\ G_t(t) \qquad \text{(DM5c)}$$

$$U_{id}(t) = \frac{V_{max,uid}\,(I_{d3}(t))\ \ G_t(t)}{K_{m,uid} +\ G_t(t)} \qquad \text{(DM5d)}$$

$$G_{tb} = \frac{U_{ii}-EGP_b+k_{tr1}\cdot G_{pb}}{k_{tr2}} \qquad \text{(DM 5e)}$$

Where $V_{max,uid}$ is described by Equation DM5f and depends on the delayed insulin signal in I_{d3}(Equation DM5g).

$$V_{max,uid}\,\left(I_{d3}(t)\right) = k_{uid1} + k_{uid2}\ \ I_{d3}(t) \qquad \text{(DM5f)}$$

$$\frac{d\,I_{d3}(t)}{dt} = \ -k_{d3}\,I_{d3}(t) +\ k_{d3}\,(I(t) - I_b)\ \ , \qquad I_{d3}(0) = 0 \quad \text{(DM5g)}$$

Finally, to impose that in the basal state EGP equals total glucose uptake, k_{uid1} is fixed according to Equation DM5h.

$$k_{uid1} = \frac{(EGP_b-U_{ii})(K_{m,uid}+G_t(0))}{G_t(0)} \qquad \text{(DM5h)}$$

Renal excretion of glucose, finally, is proportional to the height of the plasma glucose content above threshold i, as be described by Equation DM6.

$$E(t) = \begin{cases} k_e\,\left(G_p(t) - \iota\right) & if\ G_p(t) >\ \iota \\ 0 & if\ G_p(t) \leq\ \iota \end{cases} \qquad \text{(DM6)}$$

In the basal state, the **plasma insulin** content (I_p)is in a steady state with the liver insulin content (I_l). Insulin is secreted into the liver compartment, the dynamics of which are described by Equation DM7a.

$$\frac{dI_l\,(t)}{d\,t} = \ -[k_{tr4} + k_{id2}(t)]\ I_l(t) + k_{tr3}\ I_p(t) + S(t)\ , \qquad I_l(0) = I_{lb} \quad \text{(DM7a)}$$

In this equation, k_{tr4} is a parameter of insulin transportation from liver to plasma, k_{tr3} is the parameter of the reciprocal transportation from plasma to liver, and the variable $k_{id2}(t)$ describes the degradation of insulin from the liver compartment. $k_{id2}(t)$ (Equation DM7b) is a function of the hepatic extraction ratio, which, in turn, is a variable depending on insulin secretion (Equation DM7c).

$$k_{id2}(t) = \ \frac{k_{tr4}\ HE(t)}{1-\ HE(t)} \qquad \text{(DM7b)}$$

$$HE(t) = \ -k_{id3}\ S(t) + k_{id4} \qquad HE(0) = HE_b \text{(DM7c)}$$

The values of k_{tr3}, I_{lb} and k_{id4} can be calculated by imposing steady state conditions in the basal state, yielding the following equations:

$$k_{tr3} = \left(\frac{S_b}{I_{pb}} - \frac{k_{id1}}{1-\ HE_b}\ \right)\frac{1-\ HE_b}{HE_b} \qquad \text{(DM7d)}$$

$$I_{lb} = \frac{S_b +\ k_{tr3}\ I_{pb}}{k_{id2}(0)+k_{tr4}} \qquad \text{(DM7e)}$$

$$k_{id4} = \ k_{id3}S_b + HE_b \qquad \text{(DM7f)}$$

Plasma insulin (G_p) is described by Equation DM8a, including transport from the liver compartment and degradation terms.

$$\frac{dI_p(t)}{d\,t} = -[k_{tr3} + k_{id1}]\, I_p(t) + k_{tr4}\, I_l(t)\,, \qquad I_p(0) = I_{pb} \quad \text{(DM8a)}$$

$$k_{id1} = \frac{2}{5}\,\frac{S_b}{I_{pb}}\,(1 - HE_b) \qquad \text{(DM8b)}$$

$$I(t) = \frac{I_{pb}}{v_i} \qquad I(0) = I_b \quad \text{(DM8c)}$$

Insulin secretion $S(t)$ is modelled as the result of transport from the portal vein to the liver compartment (Equation DM9).

$$S(t) = \gamma \cdot I_{po}(t) \qquad \text{(DM9)}$$

While secretion into the portal vein (Equation DM10a) is described as a sum of basal, static and dynamic terms (Equations DM10b-11), where Y (Equation DM11) represents the (delayed) static term, $\kappa \cdot \dot{G}$ the dynamic term and S_b the basal term.

$$\frac{d\,I_{po}(t)}{d\,t} = -S(t) + S_{po}(t)\,, \qquad I_{po}(0) = I_{pob} \quad \text{(DM10a)}$$

$$S_{po}(t) = \begin{cases} Y(t) + \kappa \cdot \dot{G} + S_b & if\ \dot{G} > 0 \\ Y(t) + S_b & if\ \dot{G} \leq 0 \end{cases} \qquad \text{(DM10b)}$$

$$\frac{d\,Y(t)}{d\,t} = \begin{cases} -\alpha \cdot \big(Y(t) - \beta \cdot (G(t) - h)\big) & if\ \beta \cdot (G(t) - h) \geq -S_b \\ -\alpha \cdot (Y(t) + S_b) & if\ \beta \cdot (G(t) - h) < -S_b \end{cases}, \qquad Y(0) = 0 \quad \text{(DM11)}$$

Table 3. Constants and parameters of the M_{DM} model

Symbol	Unit	Value (fixed)	Value (data)	Value	Description and source
**Glucose subsystem**					
v_g	dL/kg	1.88			Glucose distribution volume
G_b	mg/dL		90		Basal plasma glucose concentration
G_{pb}	mg/kg			$G_{pb} = G_b \cdot v_g$	Basal plasma glucose content
**Endogenous glucose production**					
EGP_b			1.9		Basal endogenous glucose
k_{egp1}				$k_{egp1} = EGP_b + k_{egp2}\, G_p(0) + k_{egp3} I_{d2}(0) + k_{egp4}\, I_{po}(0)$	Maximal endogenous glucose uptake
k_{egp2}				0.0021	Parameter governing glucose feedback on EGP [1]
k_{egp3}	$\frac{mg/kg/mi}{pmol/L}$			0.009	Parameter for delayed insulin regulation of EGP [1]
k_{egp4}				0.0618	Parameter governing insulin regulation of EGP [1]
k_{d12}	min^{-1}			0.0079	Parameter of insulin delay in EGP sub-model, [1]
**Rate of appearance**					
BW	kg		78		Bodyweight, from data in [1]
θ	$-$	0.9			Proportion of glucose appearing in circulation, [7].
k_{ra1}	min^{-1}			0.057	Rate of glucose absorption from the gut, [1]
k_{ra3}	min^{-1}			0.0080	Minimal rate of stomach emptying, [1]
k_{ra4}	min^{-1}			0.0558	Maximal rate of stomach emptying, [1]
k_{ra5}	min^{-1}			$k_{ra5} = k_{ra4} = 0.0558$	Rate of transportation from Q_{sto1} to Q_{sto2} , [1] , [7]
δ	$-$			0.82	Parameter of k_{id2} , [1]
ε	$-$			0.01	Parameter of k_{id2} , [1]
ζ	mg^{-1}			$\zeta = \frac{5}{2\,D\,(1-\delta)}$	Parameter of k_{id2} , fixed according to [7] (Equation DM3f)
η	mg^{-1}			$\eta = \frac{5}{2\,D\,\varepsilon}$	Parameter of k_{id2} , fixed according to [7] (Equation DM3g)
D	mg		106		Total meal glucose content, as calculated from values of $\delta, \varepsilon, \zeta$ and η ([1]) and equations DM3f and DM3g.
**Renal excretion**					
k_e	min^{-1}			0.0005	Rate of renal excretion of glucose
i	mg/kg			339	Threshold for renal excretion of glucose

Table 3 (continued)

**Glucose uptake**					
U_{ii}	$mg/kg/min$	1			Insulin-independent glucose uptake
k_{tr1}	min^{-1}			0.065	Rate of transportation from plasma to tissue
k_{tr2}	min^{-1}			0.079	Rate of transportation from tissue to plasma
G_{tb}	mg/kg			$G_{tb} = \frac{U_{ii}-EGP_b+k_{tr1}\cdot G_{pb}}{k_{tr2}}$	Basal tissue glucose content (Equation DM5e)
k_{uid1}	$mg/kg/min$			$k_{uid1} = \frac{(EGP_b-U_{ii})(K_{m,uid}+G_{tb})}{G_{tb}}$	Basal Vmax of insulin dependent glucose uptake (Equation DM5h)
k_{uid2}	$\frac{mg/kg/mi}{pmol/L}$			0.047	Insulin-dependent term of $Vmax$
$K_{m,uid}$	mg/kg			225.59	Michaelis Menten constant for insulin-dependent glucose uptake
k_{d3}	min^{-1}			0.0331	Parameter of insulin delay in GU sub-model, [1]
**Insulin subsystem**					
k_{tr3}	min^{-1}			$k_{tr3} = \left(\frac{S_b}{I_{pb}} - \frac{k_{id1}}{1-HE_b}\right) \cdot \frac{1-HE_b}{HE_b}$	Rate of transportation from plasma to liver (Equation DM7d)
k_{tr4}	min^{-1}			0.190	Rate of transportation from liver to plasma
k_{id1}	min^{-1}			$k_{id1} = \frac{2}{5} \frac{S_b}{I_{pb}} (1 - HE_b)$	Rate of peripheral insulin degradation (Equation DM8b)
k_{id3}	min^{-1}			0.0304	Parameter of hepatic extraction, [1]
k_{id4}	$-$			$k_{id4} = k_{id3}S_b + HE_b$	Parameter of hepatic extraction, (Equation DM7f)
S_b	$pmol/kg/min$		1.55		Basal insulin secretion
κ	$\frac{pmol/kg}{mg/dL}$			2.30	Parameter of insulin secretion, [1]
α	min^{-1}			0.050	Parameter of insulin secretion, [1]
β	$\frac{pmol/kg/min}{mg/dL}$			0.11	Parameter of insulin secretion, [1]
γ	min^{-1}	0.5			Parameter of insulin secretion, [1]
HE_b	-	0.6			

Table 4. Variables, initial conditions and inputs of the M_{DM} model

Symbol	Unit	Description	Equation / value
**Variables**			
$k_{ra2}(t)$	min^{-1}	Rate of stomach emptying	$k_{ra2}\,(Q_{sto}) = k_{ra3} + \left(\frac{k_{ra4} - k_{ra3}}{2}\right)\{\tanh[\zeta(\,Q_{sto}(t) - \delta D)] - \tanh[\eta(\,Q_{sto}(t) - \varepsilon D)] + 2\}$
$k_{id2}(t)$	min^{-1}	Rate of liver insulin degradation	$k_{id2}(t) = \frac{k_{tr4}\,HE(t)}{1 - HE(t)}$
$HE(t)$	–	Hepatic extraction of insulin	$HE(t) = -k_{id3}\,S(t) + k_{id4}$
$S(t)$	$pmol/kg/min$	Insulin secretion	$S(t) = \gamma\,\cdot I_{po}(t)$
**State variables**			
G_p	$mg/\,kg$	Plasma glucose content	Equation DM1a
G_t	$mg/\,kg$	Tissue glucose content	Equation DM5a
Q_{sto1}	mg	1st stomach compartment	Equation DM3b
Q_{sto2}	mg	2nd stomach compartment	Equation DM3c
Q_{gut}	mg	Gut compartment	Equation DM3d
I_{d1}	$pmol/L$	Insulin delay (EGP)	Equation DM2c
I_{d2}	$pmol/L$	Insulin delay (EGP, following I_{d1})	Equation DM2d
I_{d3}	$pmol/L$	Insulin delay (U_{id})	Equation DM5g
I_p	$pmol/kg$	Plasma insulin content	Equation DM8a
I_l	$pmol/kg$	Liver insulin content	Equation DM7a
Ipo	$pmol/kg$	Portal vein insulin content	Equation DM10a
Y	$pmol/kg/min$	Static insulin secretion	Equation DM11

Table 4 (continued)

**Initial conditions**			
$G_p(0)$	$mg/\ kg$	Equation DM1b	$G_{pb} = G_b \cdot v_g$
$G_t(0)$	$mg/\ kg$	Equation DM5a	$G_{tb} = \frac{U_{ii} - EGP_b + k_{tr1} \cdot G_{pb}}{k_{tr2}}$
$Q_{sto1}(0)$	mg	Equation DM3b	0
$Q_{sto2}(0)$	mg	Equation DM3c	0
$Q_{gut}(0)$	mg	Equation DM3d	0
$I_{d1}(0)$	$pmol/L$	Equation DM2c	I_b
$I_{d2}(0)$	$pmol/L$	Equation DM2d	I_b
$I_{d3}(0)$	$pmol/L$	Equation DM5g	0
$I_p(0)$	$pmol/kg$	Equation DM8c	$I_{pb} = I_b \cdot v_i$
$I_l(0)$	$pmol/kg$	Equation DM7e	$I_{lb} = \frac{S_b - k_{id1}\, I_{bp}}{k_{id2}(0)}$
$Ipo(0)$	$pmol/kg$	Equation DM10 a-b	$I_{pob} = \frac{S_b}{\gamma}$
$Y(0)$	$pmol/kg$	Equation DM11	0
**Inputs**			
ins_{inf}	$pmol/kg/min$	Insulin infusion rate.	Default: 0

References

1. Dalla Man C, Rizza RA, Cobelli C. Meal simulation model of the glucose-insulin system. IEEE Trans Biomed Eng. 2007;54: 1740–1749. doi:10.1109/TBME.2007.893506

2. Roy A, Parker RS. Dynamic modeling of free fatty acid, glucose, and insulin: an extended "minimal model." Diabetes Technol Ther. 2006;8: 617–626. doi:10.1089/dia.2006.8.617

3. Jelic K, Hallgreen CE, Colding-Jørgensen M. A Model of NEFA Dynamics with Focus on the Postprandial State. Ann Biomed Eng. 2009;37: 1897–1909. doi:10.1007/s10439-009-9738-6

4. Fielding B. Tracing the fate of dietary fatty acids: metabolic studies of postprandial lipaemia in human subjects. Proc Nutr Soc. 2011;70: 342–350. doi:10.1017/S002966511100084X

5. Ruge T, Hodson L, Cheeseman J, Dennis AL, Fielding BA, Humphreys SM, et al. Fasted to Fed Trafficking of Fatty Acids in Human Adipose Tissue Reveals a Novel Regulatory Step for Enhanced Fat Storage. J Clin Endocrinol Metab. 2009;94: 1781–1788. doi:10.1210/jc.2008-2090

6. Roy A, Parker RS. A phenomenological model of plasma FFA, glucose, and insulin concentrations during rest and exercise. American Control Conference (ACC), 2010. 2010. pp. 5161–5166. doi:10.1109/ACC.2010.5530687

7. Dalla Man C, Camilleri M, Cobelli C. A system model of oral glucose absorption: validation on gold standard data. IEEE Trans Biomed Eng. 2006;53: 2472–2478. doi:10.1109/TBME.2006.883792
