# Supplementary material for: Model-Based Quantification of the Systemic Interplay between Glucose and Fatty Acids in the Postprandial State
Source: PLoS One. 2015 Sep 10;10(9):e0135665. doi: 10.1371/journal.pone.0135665 (PMC4565650; doi:10.1371/journal.pone.0135665)
Supplement: S4 File — Equations and implementation of (1) analysis of the relative control of NEFA in postprandial glucose metabolism and (2) analysis of model response to NEFA metabolism perturbation. (PDF) [file pone.0135665.s005.pdf]

S4. Model analysis.

Supplemental Data (file S4) for:

Model-based quantification of the systemic interplay between glucose and fatty acids in the postprandial state

Fianne Sips, Elin Nyman, Martin Adiels, Peter Hilbers, Peter Strålfors, Natal van Riel, Gunnar Cedersund

1. Implementation of analysis of perturbations of NEFA metabolism

To investigate the biological stability of the model in response to acute changes of NEFA metabolism, we investigated how perturbations of postprandial NEFA dynamics affected the OGTT response simulation (D_{MEAL}). Perturbation of NEFA metabolism was simulated by one of two parameter changes. In the first perturbation (which will be referred to as P_{bas}), the initial concentration of NEFA ($NEFA_0$) was multiplied by a factor of $C_{bas},$ following initialization of the model with the original value of $NEFA_0$.

$$NEFA_{0,Pbas} = NEFA_0 \cdot C_{bas} \qquad (1.1)$$

In the second perturbation, the initial concentration of NEFA was fixed to the original value of $NEFA_0$ while the value of insulin-dependent lipolysis parameter p_B was changed. The value of p_B was optimized to minimize equation 1.2 for the value of p_B; i.e. chosen such that the decrease of the NEFA concentration from the pre-prandial maximum to the nadir was diminished by a factor of C_{lip}.

$$V_{Plip}(p_{B,Clip}) = \frac{\Delta NEFA}{C_{lip}} - \Delta NEFA_{Plip}(p_{B,Clip}) \qquad (1.2)$$

In this equation, $\Delta NEFA$ is the change in NEFA concentration from the initial maximum to the postprandial minimum for the initial parameter set (from S_{Sel}), C_{lip} is the factor by which we wish to reduce $\Delta NEFA$, and $\Delta NEFA_{Plip}(p_{B,Clip})$ is the $\Delta NEFA$ in the case we have substituted the value of $p_{B,Clip}$ for the original value of p_B. Minimization of V_{Plip} for $p_{B,Clip}$ thus yields a parameter value which results in a NEFA decrease C_{lip} times as small as in the original simulation.

2. Implementation of analysis of contributions of NEFA to EGP and Uid

For calculation of the NEFA regulation to glucose uptake, we first calculate the glucose uptake regulated solely by insulin and glucose. Glucose uptake is given by Equation 2.1, as previously described in Equation 9 (main text):

$$GU(t) = U_{ii} + U_{id} = U_{ii} + \frac{V_{max,uid}\ G_t(t)}{K_{m,uid} + G_t(t)} \qquad (2.1)$$

Where U_{ii} is insulin-independent glucose uptake, U_{id} is insulin-dependent glucose uptake, G_t is the peripheral glucose concentration and $V_{max,uid}$ is a variable which depends on delayed insulin (I_{d3}) as well as delayed NEFA (N_{d1}) via Equation 2.2.

$$V_{max,uid}\ (I_{d3}(t), N_{d1}(t)) = k_{uid1} + k_{uid3}\frac{I_{d3}(t)}{N_{d1}(t)} \qquad (2.2)$$

We can thus define insulin-dependent glucose uptake while assuming NEFA constant by Equation 2.3.

$$U_{id,G,I} = \frac{V_{max,G,I}\ G_t(t)}{K_{m,uid}\ G_t(t)} \text{ with } V_{max,G,I}(t) = \left(k_{uid1} + k_{uid3}\frac{I_{d3}(t)}{N_{d1b}}\right) \qquad (2.3)$$

Herein, N_{d1b} is the initial value of N_{d1}. A NEFA regulation term cannot be computed by setting I_{d3} to $I_{d3}(0)$, as $I_{d3}(0) = 0$. This is instead calculated by subtracting the non-NEFA regulated response of equation 2.3 from the total response as is given in equation 2.1.

$$U_{id,N} = U_{id} - U_{id,G,I} \qquad (2.4)$$

Finally the contribution of NEFA to the regulation of insulin-dependent glucose uptake, $R_{N,Uid}$, is calculated by dividing the NEFA regulation term by the total response (equation 2.5).

$$R_{N,Uid} = \frac{U_{id,N}}{U_{id}} \qquad (2.5)$$

For calculation of the contribution of NEFA to EGP, the equation for EGP was first rewritten in equation 2.6:

$$EGP(t) = EGP_b + R_{Gp}(t) + R_{Id}(t) + R_{Ipo}(t) + R_N(t) \qquad (2.6)$$

Here, EGP_b is the fasting endogenous glucose production ($mg/kg/min$) and each R represents a regulation term. Specifically, we define $R_{Gp}(t) = k_{egp2}G_{pb} - k_{egp2}G_p(t)$, $R_{Id}(t) = k_{egp3}I_b - k_{egp3}I_{d2}(t)$, $R_{Ipo}(t) = k_{egp4}\ I_{pob} - k_{egp4}I_{po(t)}$ and finally $R_N(t) = k_{egp5}N_{d1}(t) - k_{egp5}N_{d1b}$.

We can then define the EGP assuming constant NEFA in equation 2.7.

$$EGP_{G,I}(t) = EGP_b + R_{Gp}(t) + R_{Id2}(t) + R_{Ipo}(t) \qquad (2.7)$$

And conversely, EGP with *only* NEFA regulation is given by equation 2.8.

$$EGP_N\ (t) = EGP_b + R_N(t) \qquad (2.8)$$

Finally, the fractional contribution of NEFA in EGP regulation is defined in equation 2.9:

$$R_{N,EGP}= \frac{R_{NEFA}(t)}{R_{Gp}(t)+R_{Id}(t)+R_{Ipo}(t)+R_{NEFA}(t)} \quad (2.9)$$

For plotting of both regulation terms, we note that the time point is chosen as the maximal absolute value, but the (more informative) relative contribution at that time point is plotted. This is necessary as the relative contribution is difficult to calculate when EGP and U_{id} are small.
