# Supplementary material for: Model-Based Quantification of the Systemic Interplay between Glucose and Fatty Acids in the Postprandial State
Source: PLoS One. 2015 Sep 10;10(9):e0135665. doi: 10.1371/journal.pone.0135665 (PMC4565650; doi:10.1371/journal.pone.0135665)
Supplement: S3 File — Zip file containing all Matlab m-files to generate figures. The zip-file includes a README file, containing an overview of the simulation files and basic instructions. (ZIP) [file pone.0135665.s004.zip › GlcNEFA/README.pdf]

README

README for Supplemental Data (file S3) for:

Model-based quantification of the systemic interplay between glucose and fatty acids in the postprandial state

Fianne Sips, Elin Nyman, Martin Adiels, Peter Hilbers, Peter Strålfors, Natal van Riel, Gunnar Cedersund

Overview of model m-files

The software for this article is provided as Matlab (MATLAB and Statistics Toolbox Release 2012a, The MathWorks, Inc., Natick, Massachusetts, United States) m-files. For parameter estimation, the software requires the optimization toolbox (we use version 6.2) to be installed. Upon unpackaging the software, the m-files should appear in the directory *GlcNEFA*. The provided files contain all datasets, models, optimization and analysis/visualization files . The files are divided into several directories; therefore to run the files from the main directory first al folders and subfolders of the folder *GlcNEFA* must be added to the path. This can be done by (a) right-clicking on the folder in Matlab and selecting "add to path" or (b) running the m-file *setDirs*, which is located in the main directory. The file *main.m* (main directory) can now be executed – note that the file is divided into subsections that may be run separately.

0. Definition of model constants

1. Importing datasets

To import a dataset for optimization, a structure is created containing all information necessary on dataset inputs, outputs, as well as labelling information -> this structure will further be referred to as the *target*. For each dataset included, an m-file is provided that

converts the digitized data (.csv files) into such a target structure (found in the *data* directory).

- MEAL generate_target_MEAL.m
- CLAMP1 generate_target_CLAMP1.m
- CLAMP2 generate_target_CLAMP2.m
- ID (independent dataset) generate_target_ID.m

We further note that in some cases, data is digitized from graphs while in others simply retained from text or tables. In the latter case, the data is copied directly into the m-file without use of a data file. The cost functions for the targets are automatically generated using the command >> `create_objFuns(T,P);`

Where T is the array of target structures to be included in the cost function and P is the array of target structures used only for plotting (P can be changed later).

2. Plotting targets (disabled)

A simple representation of an included dataset can be obtained by using the function *plot_target.* To see all inputs, outputs and basal values included in CLAMP2, for instance, the command >> plot_target(T(3)) can be used.

3. Define parameters

In this section, a structure containing all model parameters (*p*) as well as a structure (o) containing the initial parameter set for optimization (o.p0), upper and lower bounds and information necessary to translate the parameter vector used in the optimization procedure to the parameter structure used in simulations are defined.

The file *get_optim* thus contains the definition of parameter to be optimized, while *get_pars_ALL* (both found in the *parameters* directory) contains the full parameter vector.

4. Optimization

In this section, the file *optimize* is called with a settings structure to initiate a repeated optimization of the model. Here, the optimization is run repeatedly in order to generate a large set of parameters that can analyzed. In short, the settings for optimization are:

- Seed — Matlab random generator seed for the optimization. Note that each optimization run will start with a new seed – i.e. all seeds from *seed* to seed+*n* will be used.
- N — Number of times the optimization will be repeated.
- Rand — Quantity of random noise added to the initial parameter set before optimization. See *optimize.*
- Type — 'solver1' or 'solver2'
- Thres — Threshold for sum of squared errors V, for which the corresponding parameter set is saved in a text file.

The results of the optimization will be saved automatically in the directories OPT results and OPT parameters.

5. Selecting S_{ext} / S_{sel}

In this section, the file *select_results* is used to select the parameter sets used for plotting. Note that the input options of *select_results* include (a) an option to leave the minimal and maximal value of a certain parameter out of the set, and (b) a parameter used for sorting of the results, which will affect plotting order.

6. Run model for selected parameter sets

Before visualization, the following steps are taken:

- Save threshold is disabled by setting it to –Inf
- Plotting target structure P is filled and the cost functions created.
- All parameter sets in S_{ext} and S_{sel} are evaluated (with the (final) setting for output set to "all")
- The value of V_{EGP} is calculated for each set, as these are used for plotting.

7. Visualization of results

Finally, the file *visualize* is used to plot all figures included in the main text. The following files are called for final visualization.

- **Figures 3, 4, and S5**

plt_CLAMP1 and plT_CLAMP2 (analysis directory).

- **Figure 4**

plt_MEAL (analysis directory).

We note that the following text is displayed:

Maximal glucose appearance during OFTT: 12.1444 mg.

- **Figure 5**

plt_ID (analysis directory).

- **Figure 6**

plt_PERTURB (analysis directory).

- **Figure 7**

plt_CONTRIB (analysis directory).
